# LY354740 Reduces Extracellular Glutamate Concentration, Inhibits Phosphorylation of Fyn/NMDARs, and Expression of PLK2/pS129 α-Synuclein in Mice Treated With Acute or Sub-Acute MPTP

**DOI:** 10.3389/fphar.2020.00183

**Published:** 2020-02-28

**Authors:** Yang Tan, Yan Xu, Chi Cheng, Cong Zheng, Weiqi Zeng, Ji Wang, Xiaoqian Zhang, Xiaoman Yang, Jialing Wang, Xiaomei Yang, Shuke Nie, Xuebing Cao

**Affiliations:** ^1^ Department of Neurology, Union Hospital, Tongji Medical College, Huazhong University of Science and Technology, Wuhan, China; ^2^ Institute of Neuroscience, Chinese Academy of Sciences, Shanghai, China; ^3^ Department of Neurology, Renmin Hospital of Wuhan University, Wuhan, China

**Keywords:** Parkinson’s disease, 1-methyl-4-phenyl-1,2,3,6-tetrahydropyridine, metabotropic glutamate receptor, NMDA receptor, Fyn kinase, Polo-like kinase, pS129 α-synuclein

## Abstract

Glutamate overactivity in basal ganglia critically contributes to the exacerbation of dopaminergic neuron degeneration in Parkinson’s disease (PD). Activation of group II metabotropic glutamate receptors (mGlu_2/3_ receptors), which can decrease excitatory glutamate neurotransmission, provides an opportunity to slow down the degeneration of the dopaminergic system. However, the roles of mGlu_2/3_ receptors in relation to PD pathology were partially recognized. By using mGlu_2/3_ receptors agonist (LY354740) and mGlu_2/3_ receptors antagonist (LY341495) in mice challenged with different cumulative doses of 1-methyl-4-phenyl-1,2,3,6-tetrahydropyridine (MPTP), we demonstrated that systemic injection of LY354740 reduced the level of extracellular glutamate and the extent of nigro-striatal degeneration in both acute and sub-acute MPTP mice, while LY341495 amplified the lesions in sub-acute MPTP mice only. LY354740 treatment improved behavioral dysfunctions mainly in acute MPTP mice and LY341495 treatment seemed to aggravate motor deficits in sub-acute MPTP mice. In addition, ligands of mGlu_2/3_ receptors also influenced the total amount of glutamate and dopamine in brain tissue. Interestingly, compared with normal mice, MPTP-treated mice abnormally up-regulated the expression of polo-like kinase 2 (PLK2)/pS129 α-synuclein and phosphorylation of Fyn/N-methyl-D-aspartate receptor subunit 2A/2B (GluN2A/2B). Both acute and sub-acute MPTP mice treated with LY354740 dose-dependently reduced all the above abnormal expression. Compared with MPTP mice treated with vehicle, mice pretreated with LY341495 exhibited much higher expression of p-Fyn Tyr416/p-GluN2B Tyr1472 and PLK2/pS129 α-synuclein in sub-acute MPTP mice models. Thus, our current data indicated that mGlu_2/3_ receptors ligands could influence MPTP-induced toxicity, which supported a role for mGlu_2/3_ receptors in PD pathogenesis.

## Introduction

Parkinson’s disease (PD) is a common neurological disorder characterized by degeneration of dopaminergic neurons in the substantia nigra (SN) (Przedborski, 2017). Among the most widely used models of PD are those that employ toxins, especially 1-methyl-4-phenyl-1,2,3,6-tetrahydropyridine (MPTP) (Jiang and Dickson, 2018). MPTP rodent models recapitulate many aspects of the disease and are important tools to investigate the biological fundaments of PD (Meredith and Rademacher, 2011). Glutamate excitotoxicity has often been raised as the mechanism of cell death in PD (Ambrosi et al., 2014). Studies showed impaired glutamate homeostasis *in vivo* following MPTP treatment (Robinson et al., 2003; Meredith et al., 2009). Due to the compromise of mitochondrial respiration following MPTP exposure, dopaminergic neurons may not be able to cope with even low levels of glutamate stimulation and subsequent Ca^2+^ influx (Meredith and Rademacher, 2011). Therefore, mechanisms which maintain low concentrations of glutamate in PD models are essential for normal brain function.

Group II metabotropic glutamate receptors (mGlu_2_ and mGlu_3_ subtypes) are highly expressed at the pre-terminal regions of subthalamonigral synapses, where their activation can inhibit glutamate release from presynaptic membranes of these synapses (Bradley et al., 2000; Niswender and Conn, 2010; Johnson et al., 2011). mGlu_3_ subtype also presents in glial cells, especially astrocytes, which contributes to enhance glutamate uptake by astrocytes and to inhibit excess extracellular glutamate at synapses (Yao et al., 2005; Mudo et al., 2007). Early studies have demonstrated neuroprotective effects in MPTP models with several kinds of mGlu_2/3_ receptors agonists including DCG-IV and LY379268 (Matarredona et al., 2001; Battaglia et al., 2003). Battaglia’s group used LY379268 to demonstrate the protective property of mGlu_2/3_ receptors agonist in MPTP-intoxicated mice by measurement of dopamine levels and immunohistochemical analysis of TH (Battaglia et al., 2003). Matarredona’s group studied the protective effect of DCG-IV against MPP^+^-induced toxicity on rat striatal dopaminergic nerve terminals by accessing total dopamine efflux and expression of TH, GFAP and OX-42. In our experiment, LY354740, another agonist of mGlu_2/3_ receptors, was used. LY354740 and LY379268 are both highly potent and selective agonists for mGlu_2/3_, but are different in the presence of an oxygen (LY379268) or a carbon (LY354740) atom in the bicyclic ring system of the molecule and have different potencies at mGlu_3_ and mGlu_2_ (LY354740 is approximately equipotent at mGlu_2_ receptor and mGlu_3_ receptor, while LY379268 is more potent at mGlu_3_ receptor than mGlu_2_ receptor) (Monn et al., 1999). These differences in profile might account for different effects and mechanisms of these two drugs in mouse models of schizophrenia and anxiety (Menezes et al., 2013; Orlando et al., 2014). Although mGlu_2/3_ receptors have also been regarded as potential targets for the treatment of PD and levodopa-induced dyskinesias, the results were still obscure (Sebastianutto and Cenci, 2018). Activation of mGlu_2/3_ receptors by LY354740 attenuated dyskinesia development and inhibited the expression of established dyskinesia (Frouni et al., 2019), but LY379268 had no effect on reducing dyskinesia in rodent models (Rylander et al., 2009). Whether or not LY354740 has the same protective property in MPTP-intoxicated mice as LY379268 and DCG-IV is unknown and the mechanism whereby LY354740 influences neurodegeneration responses is unclear.

It is unanimously accepted that N-methyl-D-aspartate receptors (NMDARs) play a major role in excitotoxic cell death. Functional NMDARs are formed by two GluN1 and GluN2 subunits. The GluN2 subunits, mainly the GluN2A and GluN2B, as the binding sites of glutamate, are critical for several pharmacological properties of NMDAR (Riaz et al., 2015). Tyrosine phosphorylation of GluN2A and GluN2B could stabilize or enrich the expression of receptors in specific subcellular compartments, thereby potentiating the efficacies of NMDAR (especially the GluN2A phosphorylation at Tyr1325 and GluN2B phosphorylation at Tyr1472) (Lavezzari et al., 2003; Goebel et al., 2005; Zhang et al., 2008; Taniguchi et al., 2009). Fyn, a family member of Src tyrosine kinase, is located in the postsynaptic density, where it regulates phosphorylation of GluN2B (Tyr1472). Y416 phosphorylation has been widely used as a marker of Fyn activation (Ali and Salter, 2001; Salter and Kalia, 2004; Trepanier et al., 2012). Whether mGlu_2/3_ receptors ligands exert their effects on the function of postsynaptic Fyn/NMDAR is still unknown.

It has been well recognized that phosphorylated α-synuclein at Ser129 (pS129 α-synuclein) was one of the main post-translational modifications of α-synuclein in the Lewy bodies of PD brains (Anderson et al., 2006). In addition, hyperphosphorylation of α-synuclein has been linked to increased toxicity and formation of α-synuclein oligomers as well as aggregations (Samuel et al., 2016). Polo-like kinase 2 (PLK2, also known as a serum-inducible kinase, SNK) has been demonstrated as a principal contributor to pS129 α-synuclein both *in vivo* and *in vitro* (Waxman and Giasson, 2011; Basso et al., 2013; Bergeron et al., 2014). Previous research has indicated that stimuli, which produced synaptic plasticity, dramatically increased PLK2 mRNA levels (Kauselmann et al., 1999). In cultural hippocampal neurons, activation of PLK2 can be induced by glutamate (Wu et al., 2007). Whether ligands of mGlu_2/3_ receptors could influence the expression of PLK2/pS129 α-synuclein is still unknown.

In this study, we showed that pretreatment with agonist (LY354740) could reduce extracellular glutamate concentration and dopaminergic neurons loss in mice challenged with acute or sub-acute MPTP. Agonist of mGlu_2/3_ receptors might play a protective role against MPTP-induced toxicity, which might be associated with inhibition of abnormal phosphorylation of Fyn/GluN2A/2B and PLK2/pS129 α-synuclein expression. Antagonist of mGlu_2/3_ receptors (LY341495) could amplify MPTP-induced lesions, aggravate motor deficits, up-regulate abnormal expression of phosphorylated Fyn Tyr416/GluN2B Tyr1472 and PLK2/pS129 α-synuclein in sub-acute MPTP mice models. Hence, our data revealed that neurodegeneration and motor dysfunction induced by MPTP in mice might be regulated by the mGlu_2/3_ receptors ligands.

## Materials and Methods

### Animals

Male C57BL/6 wild-type mice 8–9 weeks of age, weighing 22–25 g, were purchased from HFK Bioscience Company (Beijing, China). They were housed in a barrier system under regulated temperature (21–23°C) with a 12 h light/dark cycle with food and water ad libitum. This study was carried out in accordance with the Guidelines of Laboratory Animals Ethics of Tongji Medical College, Huazhong University of Science and Technology. The proposal and research plan were approved by the Institutional Animal Care and Use Committee at Tongji Medical College, Huazhong University of Science and Technology, China.

### Chemicals

**Figure 1:**
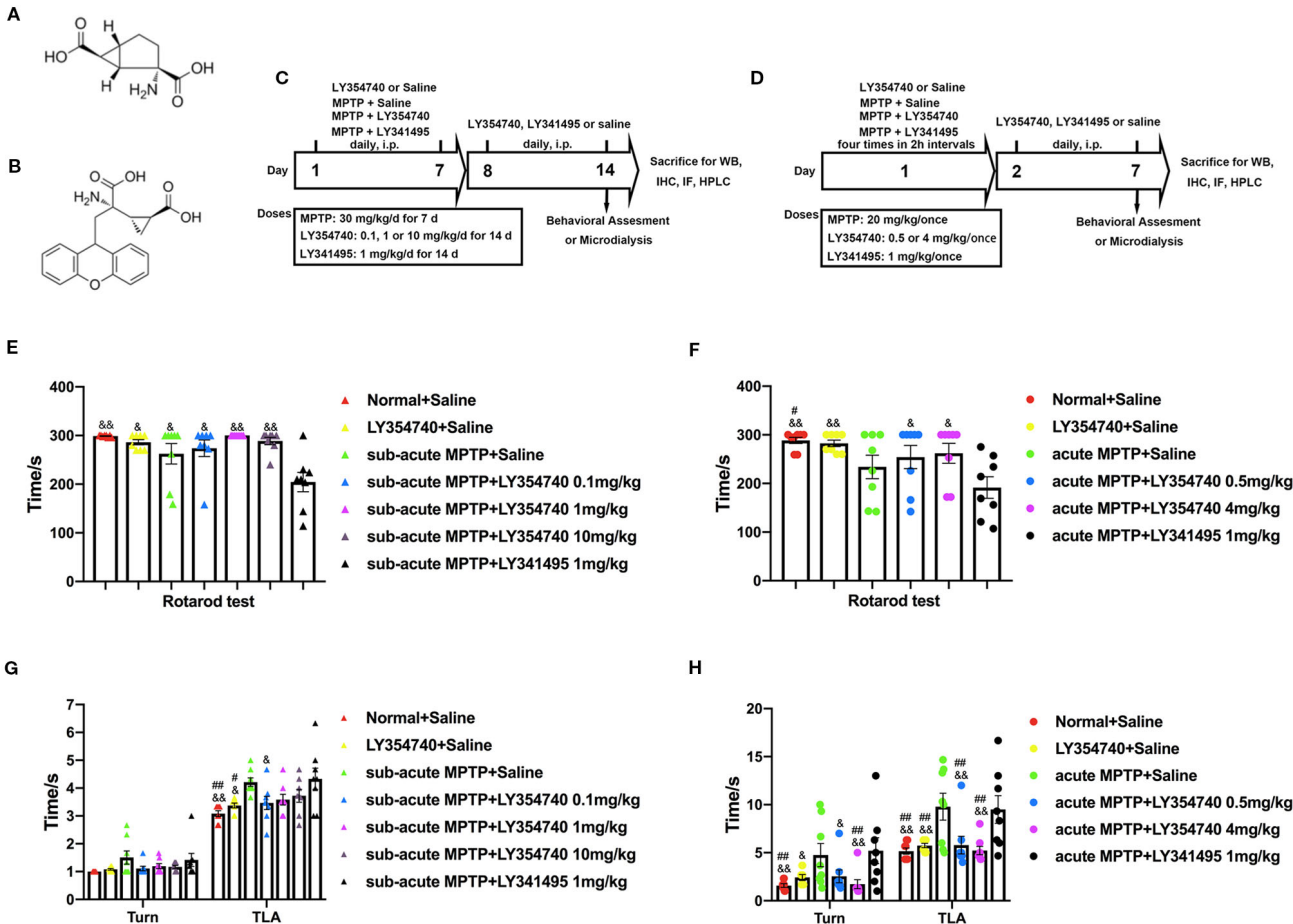
Experimental designs and the effect of chronic treatment with ligands of mGlu_2/3_ receptors on locomotor functions in acute or sub-acute 1-methyl-4-phenyl-1,2,3,6-tetrahydropyridine (MPTP)-induced Parkinson’s disease (PD) mice models. **(A)** Molecular formula of LY354740. **(B)** Molecular formula of LY341495. **(C)** Schematic representation of the sub-acute MPTP-induced PD mice model experimental scheme. **(D)** Experimental design of acute-MPTP induced PD mice models. Representative analysis of rotarod tests **(E)** and pole tests **(G)** in sub-acute MPTP mice models. Behavioral tests were performed 30 min after the last treatment of drug intervention in sub-acute MPTP mice models on the fourteenth day. ^#^p < 0.05, ^##^p < 0.01 compared with the MPTP + saline group; ^&^p < 0.05, ^&&^p < 0.01 compared with MPTP + LY341495 group. N = 8. Error bars represent SEM. Analysis of rotarod tests **(F)** and pole tests **(H)** on the seventh day of the experiment in acute MPTP mice models. Behavioral tests were performed 30 min after the last treatment of drug intervention. ^#^p < 0.05, ^##^p < 0.01 compared with the MPTP + saline group; ^&^p < 0.05, ^&&^p < 0.01 compared with the MPTP + LY341495 group. N = 8. Error bars represent SEM.

LY354740 (C_8_H_11_NO_4_, HY-18941, **Figure 1A**) and LY341495 (C_20_H_19_NO_5_, HY-70059, **Figure 1B**) were purchased from MedChemExpress (USA). They were used as a potent and selective mGlu_2/3_ receptors agonist and antagonist, respectively. Both LY354740 and LY341495 were diluted in sterile saline and sonicated at 25°C using a water bath to assist solubilization. Analytical-grade dopamine (DA) (H8502), homovanillic acid (HVA) (H1252), L-glutamic acid (95436), and 1-methyl-4-phenyl-1,2,3,6-tetrahydropyridine hydrochloride (M0896) were purchased from Sigma-Aldrich (USA). The primary antibodies were: rabbit anti-tyrosine hydroxylase (TH) (Proteintech, 25859-1-AP); mouse anti-polo-like kinase 2 (PLK2) (Santa Cruz; sc-374643); rabbit anti- pS129 α-synuclein (GeneTex; GTX54991); rabbit anti-phospho-Fyn Tyr416 (Cell Signaling Technology; CST2101); mouse anti-Fyn rabbit (Santa Cruz; sc-434); rabbit anti-GluN2A (phospho Y1325) (Abcam; ab16646); rabbit anti-GluN2B (phospho Tyr1472) (Cell Signaling Technology; CST4208); rabbit anti-GluN2A (Genetex; GTX134064); rabbit anti-GluN2B (Genetex; GTX109713); mouse anti-glial fibrillary acidic protein (GFAP) (Servicebio, GB12096); rabbit anti-β-actin (Antgene; ANT 010), rabbit anti-glyceraldehyde 3-phosphate dehydrogenase (GAPDH) (Antgene; ANT012). All appropriate secondary antibodies were purchased from Antgene: goat anti-mouse IgG (ANT019); goat anti-rabbit IgG (ANT020).

### Treatments

Prior to commencement of the experiment, all mice received behavioral training (rotarod test and pole test) for 3 days. Animals were evenly divided into different groups based on their performances. In experiment 1 (**Figure 1C**), animals were randomly divided into seven groups: Normal + Saline group, LY354740 (10 mg/kg, i.p.) + saline group, MPTP + saline group, MPTP + LY354740 (0.1, 1 or 10 mg/kg, i.p.) group and MPTP + LY341495 (1 mg/kg, i.p.) group. Mice were administered respective doses of LY354740, LY341495 or saline 30 min prior to MPTP injection (30 mg/kg/d) once a day for 7 days consecutively. Thereafter, saline, LY354740 or LY341495 were administered daily for another 7 days after the last MPTP injection.

In experiment 2, mice were randomly divided into six groups (**Figure 1D**): Normal + Saline group, LY354740 (4 mg/kg, i.p.) + saline group, MPTP + saline group, MPTP + LY354740 (0.5 or 4 mg/kg) group and MPTP + LY341495 (1 mg/kg) group. The intervention was divided into two sections. In the first section (MPTP + drug intervention), all the acute MPTP-treated mice were pretreated with saline, LY354740 or LY341495 for four times (30 min before each MPTP injection). High doses of MPTP (20 mg/kg, i.p., injected four times in 2 h intervals within 1 day) were used to establish acute-MPTP treated PD mice model. Cumulative doses of LY354740 were 2 mg/kg or 16 mg/kg and that of LY341495 was 4 mg/kg on that day. In the second section (drug intervention, without MPTP), all the mice were administered daily according to grouping (with LY354740 or LY341495 or saline, once a day) for another 6 days.

### Behavioral Assessment

Behavioral assessments were performed as our previously described and conducted at 10:00 am on each testing days (Zheng et al., 2018). Each group contained eight mice. The motor activity tests were performed by scientists who were blind to the treatment each animal received. A 30 min rest period was provided between each behavioral experiment, to alleviate animal fatigue.

#### Rotarod Test

Motor coordination was evaluated using an automated revolving rod (YLS-4C, Jinan Yiyan Technology Development Co., Ltd., China). All mice walked on the rod, which steadily accelerated from 5 rpm to 40 rpm over a period of 90 s and 40 rpm was maintained for up to 210 s. The maximum time (latency time; up to 300 s) each mouse was able to stay on the rod was recorded.

#### Pole Test

A pole with a length of 50 cm and a diameter of 1 cm was selected. The upper end of the pole was covered with a spherical protruding point with a diameter of 2 cm. Mice in each group were placed on the pole (with its head upward) at the beginning of the experiment. The base of the pole was placed in a cage, covered with a dressing. The mice would turn its head downward and descend the pole to the home cage. Time at which each mouse head turned its head down (time-turn) and the total time from the top of the pole to the bottom (time-down) were recorded as an indication of locomotion activity. Each mouse was tested three times and the average value calculated.

### Tissue Preparation

After behavioral experiments, four or five mice of each group were euthanized by decapitation under anesthesia with isoflurane for western blotting. Rapidly removed the brain and kept the tissue on ice at all times. Using a brain matrix (RWD Lifescience, China), dissected a 3.0 mm coronal slice of the striatum (anterior – posterior: +1.5 mm to -1.5 mm) and a 1.0 mm coronal slice of the midbrain (anterior – posterior: -3.0 mm to -4.0 mm) followed by finer dissection of striatum or SN with a puncher. And then, tissues were snap frozen in liquid nitrogen, then stored at -80°C until used. Three mice from each group were transcranially perfused with 4% paraformaldehyde (PFA) and brains were removed and fixed in 4% PFA at 4°C for 2 days.

### Western Blotting

A total of 40 μg proteins were electrophoresed in a 10% or 12% SDS-PAGE. Gels were run at 60 V for 3 h and then again at 100 V for 1 h. Protein was transferred to PVDF membranes (Millipore, USA). Membranes were blocked with 5% non-fat milk for 2 h at room temperature and incubated overnight at 4°C with primary antibodies against the following proteins: mouse anti-PLK2 (1:200), rabbit anti-pS129 α-synuclein (1:1000), rabbit anti-p-Fyn Tyr416 (1:1,000), mouse anti-Fyn rabbit (1:200), rabbit anti-p-GluN2A tyr1325 (1:1000), rabbit anti-p-GluN2B Tyr1472 (1:1,000), rabbit anti-GluN2A (1:1,000), rabbit anti-GluN2B (1:1,000), rabbit anti-β-actin (1:1,000), rabbit anti-GAPDH (1:2000). The day after incubation, membranes were washed three times with TBS-Tween 0.1% (TBST), then incubated with the appropriate secondary HRP-conjugated antibodies, goat anti-mouse IgG (1:3,000); goat anti-rabbit IgG (1:3,000). After washing three times, band intensities were detected with an ECL detection kit (Thermo Scientific) using the Bio-Rad imaging system. Bands intensities were quantified using Image J software. All the uncropped images of western blots are shown in **Supplementary Data**.

### Immunofluorescence and Immunohistochemistry Staining

#### Immunohistochemistry Staining

After fixation in 4% PFA for 2 days, brains (three brains per group) were processed for paraffin sectioning. Thereafter, sections were mounted on microscope slides, de-waxed using xylene, and dehydrated with ethanol at graded concentrations. Sections were baked in ethylenediaminetetraacetic acid (EDTA) (pH 9.0) antigen retrieval buffer and washed with phosphate buffered saline (PBS) (pH 7.4) three times. Then, sections were blocked with 3% bovine serum albumin (BSA) at room temperature and incubated with primary antibodies recognizing TH (1:100, Proteintech, 25859-1-AP) in a humidified chamber overnight at 4°C. Thereafter, sections were washed and incubated with biotinylated goat anti-rabbit IgG at 37°C for 30 min, then incubated with horseradish peroxidase labeled streptavidin fluid, followed by 3,3′-diaminobenzidine (DAB) solution for 5 min, counterstained with Harris hematoxylin, dehydrated, and eventually cover slipped. Images were collected using microscopy (Motic; BA310) at the same light intensity and analyzed using Image-J software.

#### Immunofluorescence Staining

Immunofluorescence staining was performed with primary antibodies recognizing PLK2 (1:50; Santa Cruz; sc-374643) or GFAP (1:200; Servicebio; GB12096) and shared the same procedure with immunohistochemistry staining prior to secondary antibody incubation. Sections were incubated with primary antibodies and washed three times. Thereafter, they were incubated in the dark with Alexa Fluor 488-coupled secondary antibodies for 50 min, followed by incubation in 4′,6-diamidino-2-phenylindole (DAPI) solution for 10 min. Images were collected using fluorescence microscopy (Olympus; BX53). Then optical density of GFAP was compared and PLK2 positive cell were counted by the image J software (Nie et al., 2015).

### Assessing Monoamines and Glutamate by High-Performance Liquid Chromatography Coupled With Electrochemical Detection (HPLC-ECD)

#### Tissue Preparation for Measurements of Monoamines and Glutamate

To evaluate the monoamines and glutamate from each group, we had another 21 mice for HPLC assessment. They were evenly divided into seven groups: normal + saline group, sub-acute MPTP + saline group, sub-acute MPTP + LY354740 (10 mg/kg, i.p.) group, sub-acute MPTP + LY341495 (1 mg/kg, i.p.) group, acute MPTP + saline group, acute MPTP + LY354740 (4 mg/kg, i.p.) group and acute MPTP + LY341495 (1mg/kg, i.p.) group. All the drug treatments were the same as before. Thirty minutes after the last i.p. injection of saline, LY354740 or LY341495, brains were rapidly removed. Brain sections containing striatum and SN were dissected as we previously described and weighed from both left and right halves, snap frozen in liquid nitrogen, then stored at −80°C for further use. Monoamine analysis was carried out as previously described (Zheng et al., 2018): tissues of 8–18 mg from the striatum were homogenized in 200 μl of 0.1 M ice-cold perchloric acid and 0.1% L-cysteine by ultrasonication. The homogenates were centrifuged at 18,000 *g* for 15 min in 4°C and supernatants were collected. For glutamate measurements, striatum and SN tissue were precipitated with 500 μl of ice-cold HPLC grade methanol, followed by centrifugation. Supernatants were collected for further derivatization. Due to the high level of glutamate in SN, 5 μl of supernatants from SN were diluted with 95 μl methanol for further assessment.

#### Microdialysis Probes and Implantation Surgery for Assessing Extracellular Glutamate Concentration

To further determine the influence of LY354740 on extracellular glutamate concentration, we performed microdialysis *in vivo*. We had another 12 mice and they were evenly divided into four groups (three animals per group): sub-acute MPTP + saline group, sub-acute MPTP + LY354740 (10 mg/kg, i.p.) group, acute MPTP + saline group, acute MPTP + LY354740 (4 mg/kg, i.p.) group. All the drug treatments were the same as before. Surgical and microdialysis procedures were conducted as previously described (Zheng et al., 2018). Male C57BL/6 mice, weighing 25–30 g, were anesthetized with isoflurane (induction 3%, maintenance 1.5%). During surgery, mice were placed on a heating pad to avoid hypothermia. According to the atlas of Paxinos (Paxinos, 2013), a guide cannula (Eicom/Japan; AG-4) was implanted at the following stereotactic coordinates: A/P -3.1 mm, M/L -1.2 mm from bregma and D/V -3.8 mm from dural surface. Thereafter, the cannula was fastened to the skull surface by dental cement and three stainless steel screws (RWD/China; 62514; 1 mm × 2 mm). Cap of the cannula (Eicom/Japan; A-D-4) was inserted to prevent obstruction and possible infection. After surgery, mice were allowed 4 days to recover and antibiotics such as penicillin were administered. Thereafter, mice were evenly divided into four groups: acute MPTP/saline group, acute MPTP/LY354740 (4 mg/kg) group, sub-acute MPTP/saline group, sub-acute MPTP/LY354740 (10 mg/kg) group. On the last day of intervention, a dialysis probe (Eicom/Japan; A-Z-4-1; 1 mm dialyzing membrane) was implanted through the guide cannula into SN. Briefly, the tip of the guide cannula was positioned above the SN and the dialysis probe lowered so that the probe (1 mm) reached the most ventral aspect of the SN. The microdialysis pump (LongerPump/China; LSP02-1B) was connected to the probe *via* Teflon tubings (Eicom/Japan; JT-10-80 and JB-30) and artificial cerebrospinal fluid (aCSF; 140 mM NaCl, 3.0 mM KCl, 1.2 mM CaCl2, 1.0 mM MgCl_2_-6H_2_O, 1.2 mM Na_2_HPO_4_-12H_2_O, 0.27 mM NaH_2_PO_4_-2H_2_O, 7.2 mM D-glucose, pH 7.3–7.5) was perfused at a rate of 2 μl/min. After stabilization period of 1 h, samples were collected for 30 min. Then, LY354740 were given as previously described, 30 min later, samples were collected for 30 min and stored at -80°C. Mean probe recovery was 13.79%. At the end of experiment, animals were sacrificed, and the correct placement of probes was verified histologically.

#### Analysis by HPLC-ECD

**Figure 2:**
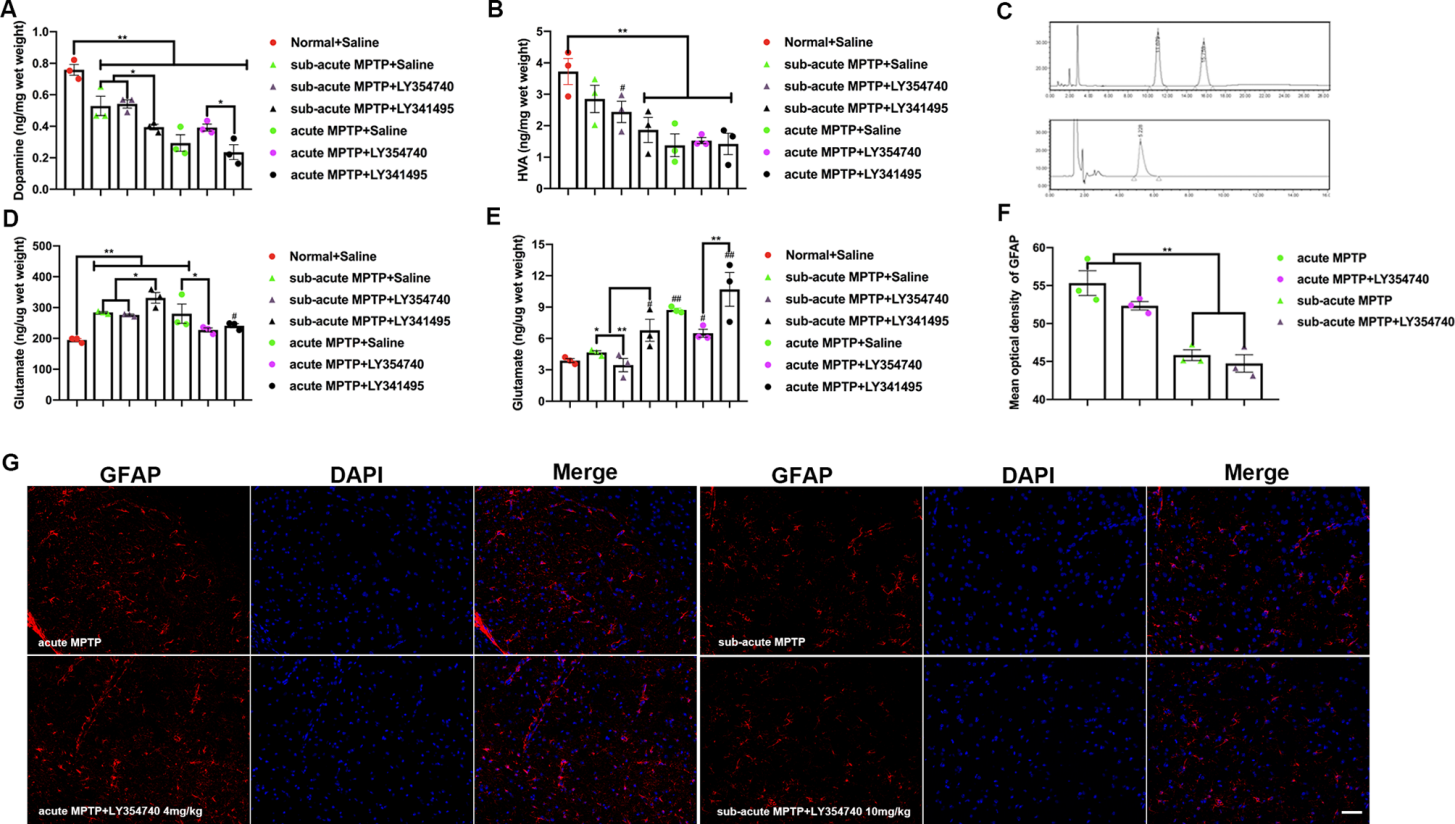
Ligands of mGlu_2/3_ receptors regulate the levels of dopamine (DA), homovanillic acid (HVA), and glutamate in 1-methyl-4-phenyl-1,2,3,6-tetrahydropyridine (MPTP) treated mice. Levels of DA **(A)** and HVA **(B)** detected in the striatum and levels of glutamate detected in the **(D)** substantia nigra (SN), **(E)** striatum. Data are expressed as ng/µg or ng/mg wet weight tissue and represent the mean ± SEM (n = 3). ^#^p < 0.05, ^##^p < 0.01 compared to the normal + saline group; ^*^p < 0.05, ^**^p < 0.01. **(C)** Sample chromatogram of standard solution. Peaks in order: DA (11 min), HVA (15 min). Illustration of the glutamate standard peak (5 min). **(F**, **G)** Optical density analysis and immunofluorescence of glial fibrillary acidic protein (GFAP) in SN. Scale bar = 50 µm. ^**^p < 0.01. N = 3. Error bars represent SEM.

Mixture standards of monoamines (DA and HVA; 1 mg/ml, in 0.1 M perchloric acid and 0.1% L-cysteine) were prepared by diluting stock solutions with the mobile phase into 500, 166.7, 55.6, 6.2 and 2.1 ng/ml. Standard of glutamate (1 mg/ml, in HPLC grade methanol) for tissue homogenate samples was diluted with the mobile phase into 750, 250, 27.7, and 3.09 ng/μl. Standard of glutamate (1 mg/ml, in HPLC grade methanol) for microdialysis samples was diluted with the mobile phase into 200, 100, 20, 2 pg/μl. Sample chromatograms of standard solution were shown in **Figure 2C**. Upper chromatogram shows the DA peak (11 min) and HVA peak (15 min). Under illustration showed chromatogram of the glutamate standard peak (5 min). For glutamate assessment, samples were precolumn derivatized with o-phthalaldehyde (OPA) reagent solution. The derivatizing agent was prepared according to previous reports (Smith and Sharp, 1994; Lehner, 2015): 2.2 mg of OPA was dissolved in 0.05 ml of 1 M sodium sulfite, 0.05 ml of absolute ethanol and 0.9 ml of 0.4 M sodium tetraborate buffer (adjusted to pH 10.4 with 5 M sodium hydroxide). The reagent was made up in a darkened vial and stored away from light at 4°C for 8 h. Standards (50 μl) and samples were precolumn derivatized with 5 μl of derivatizing agent at room temperature for 2 min before injection into the HPLC system. All samples were injected into the Waters 510 HPLC system and pumped through the column (Waters Spherisorb 5 μm ODS1, 4.6 mm × 25 cm). The column temperature was set at 30°C for monoamines and 20°C for glutamate analysis. Detection of compounds was performed with Waters 2465 Electrochemical Detector. The electrode potential was 800 mV and the mobile phase (100 mM sodium acetate, 85 mM citric acid, 3 mM sodium heptane sulfonate, 0.2 mM EDTA, and 8% methanol) was at a flow rate of 1 ml/min.

### Statistical Analysis

To evaluate protein levels among groups (p-Fyn tyr416, p-GluN2A Tyr1325, p-GluN2B Tyr1472, PLK2, and pS129 α-synuclein), all variables above were first assessed by the Shapiro-Wilk test (test of normality), Box’s test (test of covariance matrices) and Levene’s test (test of equality of error variances). Multivariate analysis of variance (MANOVA) was conducted using Pillai’s trace to reveal the different expressions of these proteins. We found that there were significant differences in experiment 1 (Pillai’s trace V = 2.26, p < 0.05) and in experiment 2 (Pillai’s trace V = 2.191, p < 0.01).

Then, one-way analysis of variance (ANOVA) followed by least significant difference (LSD) post-hoc comparisons for multiple groups comparisons were conducted (more than two groups) (Zheng et al., 2018). Paired samples T test for comparing extracellular glutamate concentrations before and after LY354740 injection (**Table 1** for details). Independent samples T test for comparing extracellular glutamate concentration between two independent groups (**Table 1** for details). A value of p < 0.05 indicated significance. All data were presented as the mean ± standard error of mean (SEM). Data were analyzed using SPSS Statistics 22.0.

**Table 1:** Extracellular glutamate determination (pg/μl).

	Saline	LY354740			
		Before	After	Correlation	Differences
Acute MPTP	510.25 ± 39.58	337.81 ± 29.10^#^	225.88 ± 23.93^##^	0.999^*^	121.93 ± 5.34^**^
Sub-acute MPTP	268.98 ± 8.20^$^	216.35 ± 6.16^##^	154.42 ± 10.83^##^	0.998^*^	61.94 ± 4.70^**^ ^$$^

Differences: changes of glutamate concentration before and after LY354740 administration. All values are means ± SEM. N = 3 per group.

* p < 0.05; ^**^p < 0.01; (Paired samples T test).

$ p < 0.01 vs acute MPTP/saline group (Independent samples T test).

$$ p < 0.01 vs differences of acute MPTP/LY354740 group (Independent samples T test).

# p < 0.05, ^##^p < 0.01 vs. corresponding MPTP/saline group (Independent samples T test).

## Results

### Effects of mGlu_2/3_ Receptors Ligands on MPTP-Induced Behavioral Impairments in Acute and Sub-Acute MPTP Mice Models

We first examined the functional effects of mGlu_2/3_ receptors ligands in acute and sub-acute MPTP mice models (**Figure 1**). Motor performances were assessed by the rotarod test and pole test. In the rotarod test of sub-acute mice models (**Figure 1E**), no obvious differences were detected among the normal + saline group, LY354740 + saline group and sub-acute MPTP + LY354740 group. However, latency times in sub-acute MPTP-mice treated with LY341495 (1 mg/kg) significantly decreased compared with the normal + saline group (p < 0.01), sub-acute MPTP + LY354740 (1 or 10 mg/kg) group (p < 0.01), LY354740 + saline group (p < 0.05), sub-acute MPTP + saline group (p < 0.05), and sub-acute MPTP + LY354740 (0.1 mg/kg) (p < 0.05). In the rotarod test of acute mice models (**Figure 1F**), latency time of acute-MPTP + saline group was obviously decreased when compared with the normal + saline group (p < 0.05). Latency times in acute MPTP-mice treated with LY341495 (1 mg/kg) significantly decreased compared with sub-acute MPTP + LY354740 (0.5 or 4 mg/kg) group (p < 0.05), normal + saline group (p < 0.01) and LY354740 + saline group (p < 0.01). No obvious differences were detected between acute-MPTP + saline group and acute-MPTP + LY341495 group.

We further assessed pole tests. In sub-acute MPTP-treated experiment (**Figure 1G**), no significant differences were detected among treatment groups in time-turn (Turn). However, a significant longer time-down (TLA) was spent by the MPTP + saline group and MPTP + LY341495 group compared with the normal + saline group (both p < 0.01) and LY354740 + saline group (both p < 0.05). In addition, MPTP mice pretreatment with LY354740 (0.1 mg/kg) were performed better than that of the MPTP + LY341495 group (p < 0.05). In acute MPTP-treated group (**Figure 1H**), both MPTP + saline group and MPTP + LY341495 group exhibited longer times (Turn) than normal + saline group and MPTP + LY354740 (4 mg/kg) group (p < 0.01). Moreover, these two groups also exhibited longer times (TLA) than all the other groups (p < 0.01). In pole tests, no obvious treatment effect was found between MPTP + saline group and MPTP + LY341495 group or among the normal + saline group, LY354740 + saline group and MPTP + LY354740 group. All these data revealed that systemic injection of mGlu_2/3_ receptors agonist alleviate MPTP induced motor deficits, detected by pole tests, especially in acute MPTP-treated mice. mGlu_2/3_ receptors antagonist might aggravate locomotor function induced by MPTP, detected by rotarod test, especially in sub-acute MPTP-treated mice.

### Total DA, HVA, and Glutamate Concentrations in Homogenates: *Ex Vivo* Measurements Using the HPLC-ECD Method

To characterize the effects of mGlu_2/3_ receptors on MPTP treated mice, levels of glutamate, DA and its main metabolites (HVA) were studied. DA levels from homogenates of striatum were measured (**Figure 2A**). As expected, all the mice treated with MPTP reduced DA levels compared with normal + saline group (p < 0.01). The antagonist LY341495 amplified the reduction in striatal DA levels induced by sub-acute MPTP treatment (p < 0.05). Higher levels of DA were found in acute-MPTP mice treated with LY354740, compared with mice treated with LY341495 (p < 0.05). We observed a rising trend of dopamine concentration in the acute (p = 0.096) or sub-acute (p = 0.212) MPTP mice co-administrated with LY354740 compared with corresponding MPTP + saline mice, but no significant difference was found between them. The HVA in striatum was also measured (**Figure 2B**). Compared with the normal + saline group, all mice treated with acute MPTP (p < 0.01) and mice from the sub-acute MPTP + LY341495 group (p < 0.01) or sub-acute MPTP + LY354740 group (p < 0.05) expressed a lower level of HVA.

With nigro-striatal dopaminergic depletion, glutamatergic projections become overactive and result in increased glutamatergic contents in basal ganglia circuitry (Lindefors and Ungerstedt, 1990; Zheng et al., 2019). The glutamatergic contents of brain tissue homogenate could partially indicate the extents of glutamatergic neurotransmission and nigro-striatal degeneration. Glutamate levels from homogenates of striatum and SN were also measured. Compared with the normal + saline group (**Figure 2D**), we found an increase in glutamate content in SN of all mice treated with sub-acute MPTP (p < 0.01), mice treated with acute-MPTP + saline (p < 0.01) and mice treated with acute-MPTP + LY341495 (1 mg/kg) (p < 0.05). In addition, LY341495 (1 mg/kg) significantly up-regulated the level of glutamate in sub-acute MPTP-treated mice compared with the other two groups (sub-acute MPTP + LY354740 and sub-acute MPTP + saline; p < 0.05). Moreover, LY354740 (4 mg/kg) significantly down-regulated level of glutamate in acute MPTP-treated mice, compared with acute-MPTP + saline group (p < 0.05). No significant difference was found between mice treated with acute-MPTP + LY354740 (4 mg/kg) and normal + saline group.

We also explored the level of glutamate in striatum (**Figure 2E**). LY341495 treatment (1 mg/kg) significantly increased striatal glutamate level compared with other mice treated with LY354740 (4 mg/kg in acute-MPTP group or 10 mg/kg in sub-acute MPTP group; p < 0.01). Significant difference was found between sub-acute MPTP + saline group and sub-acute MPTP + LY341495 group (p < 0.05). Although we observed a rising trend of glutamate concentration after LY341495 treatment, no significant difference was found between acute MPTP + saline group and acute-MPTP + LY341495 group (p = 0.138). In addition, sub-acute MPTP mice treated with LY341495 (p < 0.05), acute-MPTP mice treated with saline (p < 0.01), acute-MPTP mice treated with LY341495 (p < 0.01) or acute-MPTP mice treated with LY354740 (p < 0.05) evidently increased the glutamate level, compared with normal + saline group.

### Measurements of Glutamate in Mice SN *In Vivo* by HPLC-ECD

To further determinate the influence of LY354740 on extracellular glutamate concentration, we performed microdialysis *in vivo* (**Table 1**). Chronic injection with LY354740 showed a significant decrease in extracellular glutamate levels in SN compared to the corresponding MPTP/saline group (p < 0.05 in acute MPTP group; p < 0.01 in sub-acute MPTP group). Meanwhile, basal levels of extracellular glutamate concentration in the acute MPTP/saline group are much higher than mice treated with sub-acute MPTP/saline (p < 0.01). Extracellular glutamate concentrations before and after LY354740 injection were analyzed by paired samples T test. After injection of LY354740 for 30 min, glutamate concentrations significantly decreased from 337.81 ± 29.10 pg/μl to 225.88 ± 23.93 pg/μl in acute-MPTP/LY354740 mice (correlation = 0.999, p < 0.05; differences = 121.93 ± 5.34 pg/μl, p < 0.01). Significant paired differences were also found in sub-acute MPTP/LY354740 mice before (216.35 ± 6.16 pg/μl) and after (154.42 ± 10.83 pg/μl) LY354740 injection (correlation = 0.998, p < 0.05; differences = 61.94 ± 4.70 pg/μl, p < 0.01). Changes of glutamate concentration (before and after LY354740) between the acute and sub-acute MPTP group were analyzed by independent samples T test. Interestingly, although the dose of LY354740 in sub-acute MPTP mice was higher than that in acute-MPTP mice, more apparent changes in the extracellular concentration of glutamate was found in mice treated with acute doses of MPTP (p < 0.01). Basal extracellular glutamate concentration was consistent with expression of astrocytes (Han et al., 2008). This might be related to different amounts of astrocyte (**Figures 2F, G**). We found that mice pretreated with sub-acute MPTP/LY354740 contained less astrocytes than mice pretreated with acute MPTP/LY354740 (p < 0.01). Thus, more astrocytes might be activated for more extracellular glutamate uptake in acute MPTP/LY354740 mice than that in sub-acute MPTP/LY354740 mice.

### Impacts of mGlu_2/3_ Receptors Ligands on MPTP-Induced Degeneration of Nigral-Striatal Dopaminergic Neurons in Sub-Acute MPTP Mice Models

**Figure 3:**
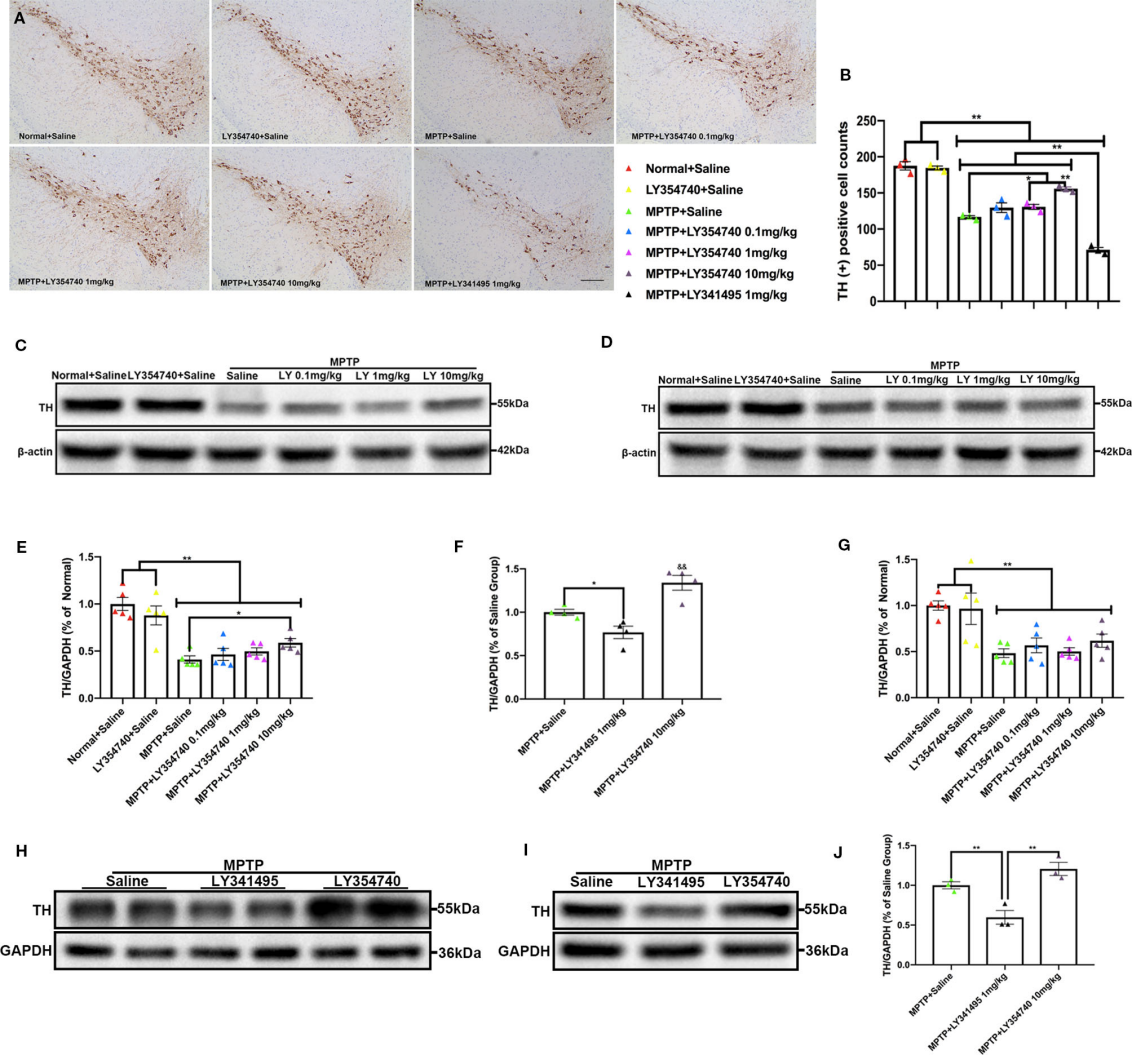
Ligands of mGlu_2/3_ receptors influence expression of tyrosine hydroxylase (TH) in striatum and substantia nigra (SN) of sub-acute 1-methyl-4-phenyl-1,2,3,6-tetrahydropyridine (MPTP)-treated mice. **(A**, **B)** Immunohistochemical images and corresponding TH positive cell counts in SN from each group. Scale bar = 100 μm. All data were expressed as % of the normal + saline group. ^*^p < 0.05, ^**^p < 0.01. *N* = 3 per group. Error bars represent SEM. **(C, E, F, H)** Immunoblots present the levels of TH in SN of each group. ^*^ *p* < 0.05, ^**^p < 0.01; ^&&^p < 0.01 vs. the other the two groups. N = 5 per group in **(E)**; N = 4 per group in **(F)**. **(D, G, I, J)** Relative quantifications of expression of TH in striatum of each group were presented. ^**^p < 0.01. N = 5 per group in **(G)**; N = 3 per group in **(J)**. The band intensity of particular target proteins was normalized to glyceraldehyde 3-phosphate dehydrogenase (GAPDH) or β-actin level. Data in **(E, G)** were expressed as % of normal + saline group. Data in **(F, J)** were expressed as % of the MPTP + saline group. Error bars represent SEM.

In the pilot test, LY354740 was administered for 7 days at the dose of 4 mg/kg in sub-acute MPTP mice and no obvious protective effect of LY354740 was found (data not shown). Consequently, we extended the time of intervention to 14 days and escalated the dose of LY354740 to 10 mg/kg. We stained brain slices of SN with TH (**Figures 3A, B**) and found that the numbers of TH-positive cells decreased in all the sub-acute MPTP-treated mice compared with the normal + saline group and the LY354740 + saline group (p < 0.01). Sub-acute MPTP-mice treated with LY354740 at the dose of 1 (p < 0.05) or 10 mg/kg (p < 0.01) attenuated TH (+) cells reduction compared with the sub-acute MPTP + saline group. In addition, compared with all the other groups of sub-acute MPTP-mice (treated with saline or LY354740), fewer TH-positive cells were counted in sub-acute MPTP-mice treated with LY341495 (p < 0.01). All the above data were paralleled by western blot analysis of TH in SN lysate (**Figures 3C, E, H, F**). We also quantified the level of TH in striatum using western blot analysis. Although a statistical significance was noted in the expression levels of TH between all the sub-acute MPTP-treated groups and the normal + saline group (p < 0.01; **Figures 3D, G**), no significant difference was found among the sub-acute MPTP + saline group and sub-acute MPTP + LY354740 groups. In addition, no difference was found between the normal + saline group and LY354740 + saline group in both SN and striatum. Interestingly, sub-acute MPTP mice treated with LY341495 expressed lower TH than the sub-acute MPTP + saline group and sub-acute MPTP + LY354740 (10 mg/kg) group in striatum (p < 0.01; **Figures 3I, J**). Overall, in sub-acute MPTP mice models, LY354740 might dose-dependently provided protection against MPTP toxicity to some extent, while LY341495 could amplify neurodegeneration induced by lower toxic doses of MPTP.

### Influences of mGlu_2/3_ Receptors Ligands on Phosphorylation of Fyn/NMDARs, PLK2, and pS129 α-Synuclein in Sub-Acute MPTP Mice Models

**Figure 4:**
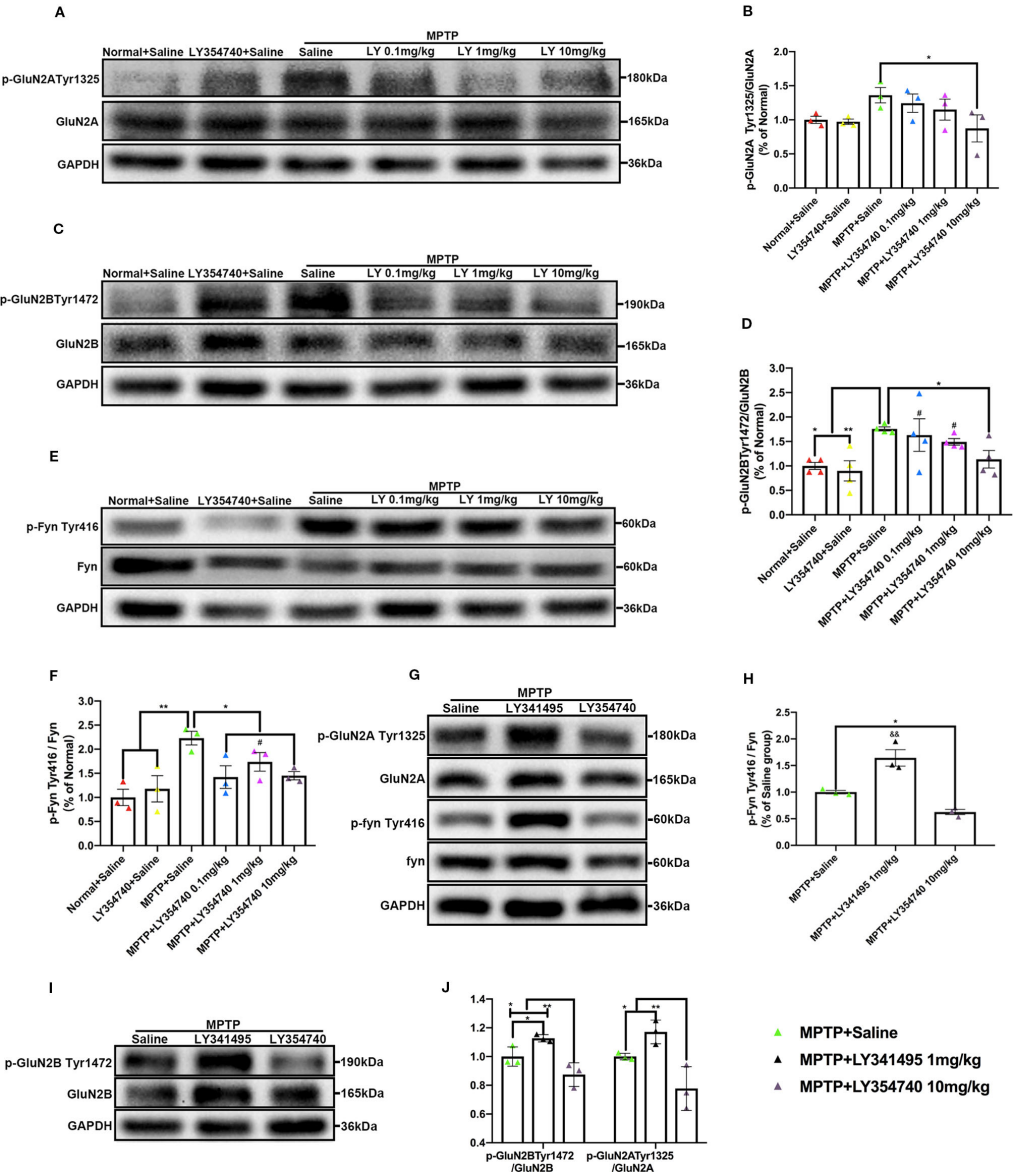
Ligands of mGlu_2/3_ receptors influence expression of phosphorylated Fyn and subunits of N-methyl-D-aspartate receptors (NMDAR) in substantia nigra (SN) in sub-acute 1-methyl-4-phenyl-1,2,3,6-tetrahydropyridine (MPTP)-treated mice. Western blot analysis showed the levels of p-GluN2A tyr1325 **(A)**, p-GluN2B Tyr1472 **(C)** and p-Fyn Tyr416 **(E)** in SN of each group. Quantification of ratios of p-GluN2A tyr1325/GluN2A **(B)**, p-GluN2B Tyr1472/GluN2B **(D)**. ^*^p < 0.05, ^**^p < 0.01; ^#^p < 0.05 vs. LY354740 + saline group. N = 3 per group in **(B)**; N = 4 per group in **(D)**. Quantification of the ratio of p-Fyn Tyr416/Fyn **(F)** of each group. ^*^p < 0.05, ^**^p < 0.01; ^#^p < 0.05 vs. the normal + saline group. N = 3. Relative band intensities were calculated as a ratio of the phosphorylated protein to total protein. The data above were expressed as % of the normal + saline group and error bars represent SEM. Western blot analysis showed the levels of p-GluN2A tyr1325, p-Fyn Tyr416 **(G)** and p-GluN2B Tyr1472 **(I)** in SN of each group. **(J)** Quantification of ratios of p-GluN2A tyr1325/GluN2A and p-GluN2B Tyr1472/GluN2B of each group. ^*^p < 0.05, ^**^p < 0.01. N = 3. **(H)** Quantification of ratios of p-Fyn Tyr416/Fyn of each group. ^*^p < 0.05; ^&&^p < 0.01 vs. the other two groups. N = 3. Relative band intensities were calculated as a ratio of the phosphorylated protein to total protein. Data in **(H, J)** were expressed as % of the saline group and error bars represent SEM.

We showed that LY354740 could regulate extracellular glutamate concentration, but whether they could influence phosphorylation of postsynaptic NMDA receptors, mainly the GluN2 subunits where the glutamate binds to, was still unknown. We conducted western blotting analysis to examine the phosphorylation of Fyn/NMDA receptors. We observed a rising trend of p-GluN2A Tyr1325 in the sub-acute MPTP + saline group compared with the normal + saline group (p = 0.069) or LY354740 + saline group (p = 0.052); although no significant difference was found among them (**Figures 4A, B**). In addition, sub-acute MPTP mice treated with LY354740 showed a downtrend of this phosphorylated protein, especially at a dose of 10 mg/kg, which had a noticeable difference compared with the MPTP + saline group (p < 0.05). We also discovered that the GluN2B was more prominently phosphorylated in the sub-acute MPTP + saline group than the LY354740 + saline group (p < 0.01) and the normal + saline group (p < 0.05; **Figures 4C, D**), whereas the level of the phosphorylated protein was reduced by LY354740 of three doses. An apparent difference was found between the MPTP + saline group and the MPTP + LY354740 (10 mg/kg) group (p < 0.05; **Figures 4C, D**). As we expected, sub-acute MPTP treatment abnormally up-regulated expression of p-Fyn Tyr416 compared with the normal + saline group and the LY354740 + saline group (p < 0.01; **Figures 4E, F**), while LY354740 (0.1 or 10 mg/kg) down-regulated this abnormal expression, with a significant difference compared with the MPTP + saline group (p < 0.05; **Figures 4E, F**). Overall, no difference was found between the normal + saline group and the LY354740 + saline group. We also examined the impact of LY341495 in sub-acute MPTP mice models. We found that mice treated with MPTP + LY341495 exhibited much higher expressions of p-Fyn Tyr416 (p < 0.01, compared with the other two groups; **Figures 4G, H**) and p-GluN2B Tyr1472 (p < 0.05, compared with MPTP + saline group; p < 0.01, compared with the MPTP + LY354740 group; **Figures 4I, J**). Although the level of p-GluN2A Tyr1325 showed a rising trend, no significant difference was found between the MPTP + LY341495 group and the MPTP + saline group (**Figures 4G, J**).

**Figure 5:**
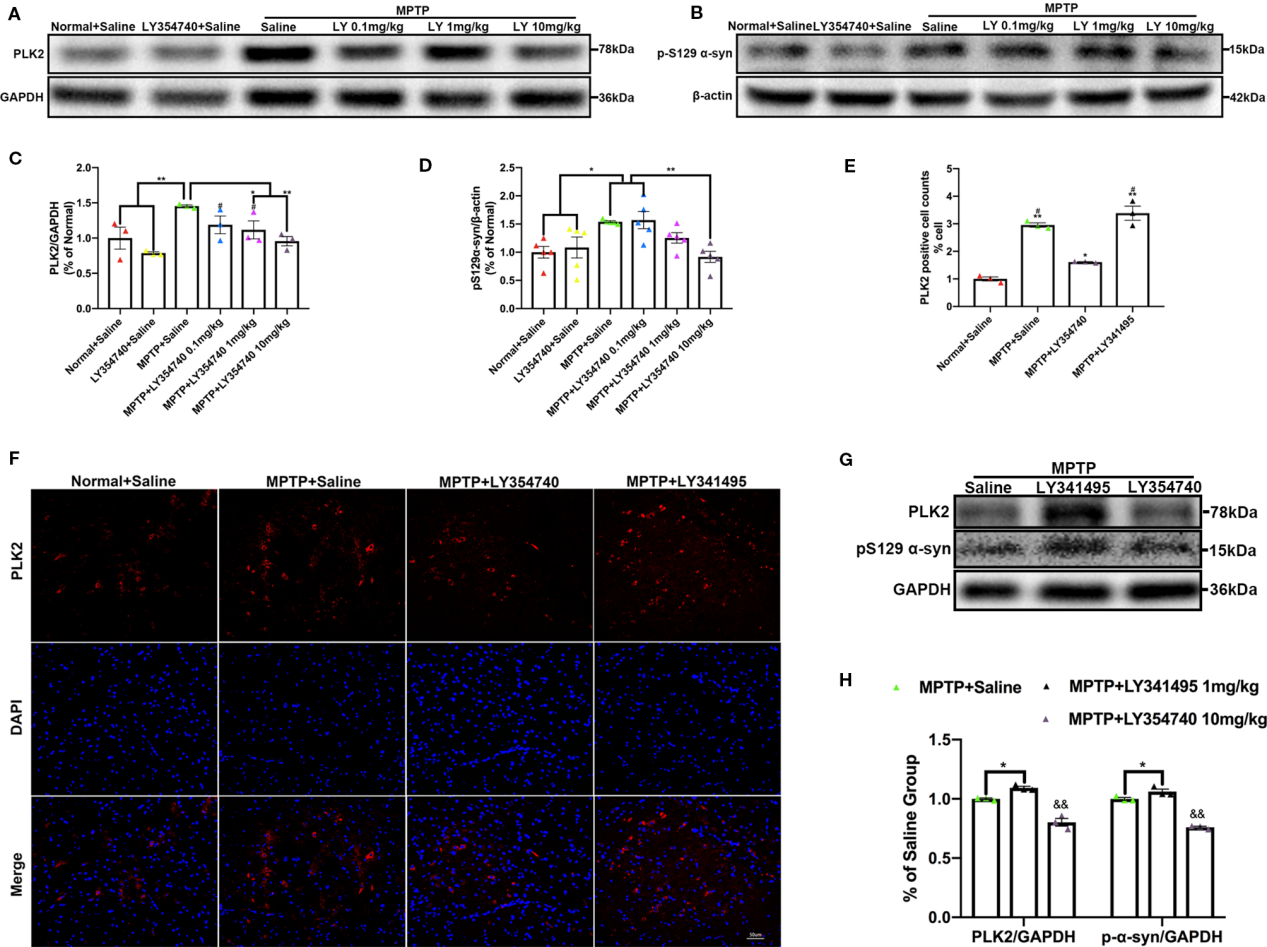
Ligands of mGlu_2/3_ receptors regulate expression of polo-like kinase 2 (PLK2) and phosphorylated α-synuclein at Ser129 in substantia nigra (SN) of sub-acute 1-methyl-4-phenyl-1,2,3,6-tetrahydropyridine (MPTP)-induced Parkinson’s disease (PD) mice model. Western blots represent the expression of PLK2 **(A)** and pS129 α-synuclein **(B)** in SN of each group. The band intensity of particular target proteins was normalized to glyceraldehyde 3-phosphate dehydrogenase (GAPDH) or β-actin level **(C, D)**. ^*^ *p* < 0.05, ^**^ *p* < 0.01; ^#^ *p* < 0.05 vs. LY354740 + saline group. N = 3 in **(C)**; N = 4 in **(D)**. Data were expressed as % of the normal + saline group and shown as the mean ± SEM. Immunoblots **(G)** and corresponding histograms **(H)** show expression of PLK2 and pS129 α-synuclein in SN. ^&&^ *p* < 0.01 compared with other two groups; ^*^ *p* < 0.05. N = 3. Data were expressed as % of the MPTP + saline group and shown as the mean ± SEM. **(E, F)** Immunofluorescence and analysis of PLK2 positive cell counts in SN. Scale bar = 50 µm. ^*^ *p* < 0.05, ^**^ *p* < 0.01 compared with Normal+Saline group; ^#^ *p* < 0.05 compared with MPTP+LY354740 group. N = 3. Data were shown as the mean ± SEM.

We further examined the levels of toxic forms of α-synuclein in the SN of sub-acute MPTP mice. We found that compared with the normal and LY354740 + saline groups, the levels of PLK2 (p < 0.01) and pS129 α-synuclein (p < 0.05) of the MPTP + saline group were significantly higher (**Figures 5A–D**). The levels of certain proteins were reduced by LY354740 of three doses, especially the highest dose of 10 mg/kg, compared with the MPTP + saline group (p < 0.01, **Figures 5A–D**). No significant difference was found among the normal + saline group, LY354740 + saline group and the MPTP + LY354740 (10 mg/kg) group (**Figures 5A–D**). Meanwhile, LY341495 significantly up-regulated the expressions of PLK2/pS129 α-synuclein compared with the MPTP + saline group (p < 0.05) and MPTP+LY354740 (10 mg/kg) group (p < 0.01; **Figures 5G, H**). We also performed immunofluorescence of PLK2 in the SN (**Figures 5E, F**). The results showed that PLK2 evenly distributed within the neurons. Compared with normal mice, mice in MPTP + Saline group (p < 0.01), MPTP + LY354740 group (p < 0.05), and MPTP + LY341495 group (p < 0.01) showed an elevation of the number of PLK2 positive cells, indicating increased expression of PLK2. LY354740 (10 mg/kg) had a fewer and dispersive distribution of PLK2 (p < 0.05) compared with the MPTP + saline and MPTP + LY341495 group.

### Effects of mGlu_2/3_ Receptors Ligands on MPTP-Induced Degeneration of Nigral-Striatal Dopaminergic Neurons in Acute MPTP Mice Models

**Figure 6:**
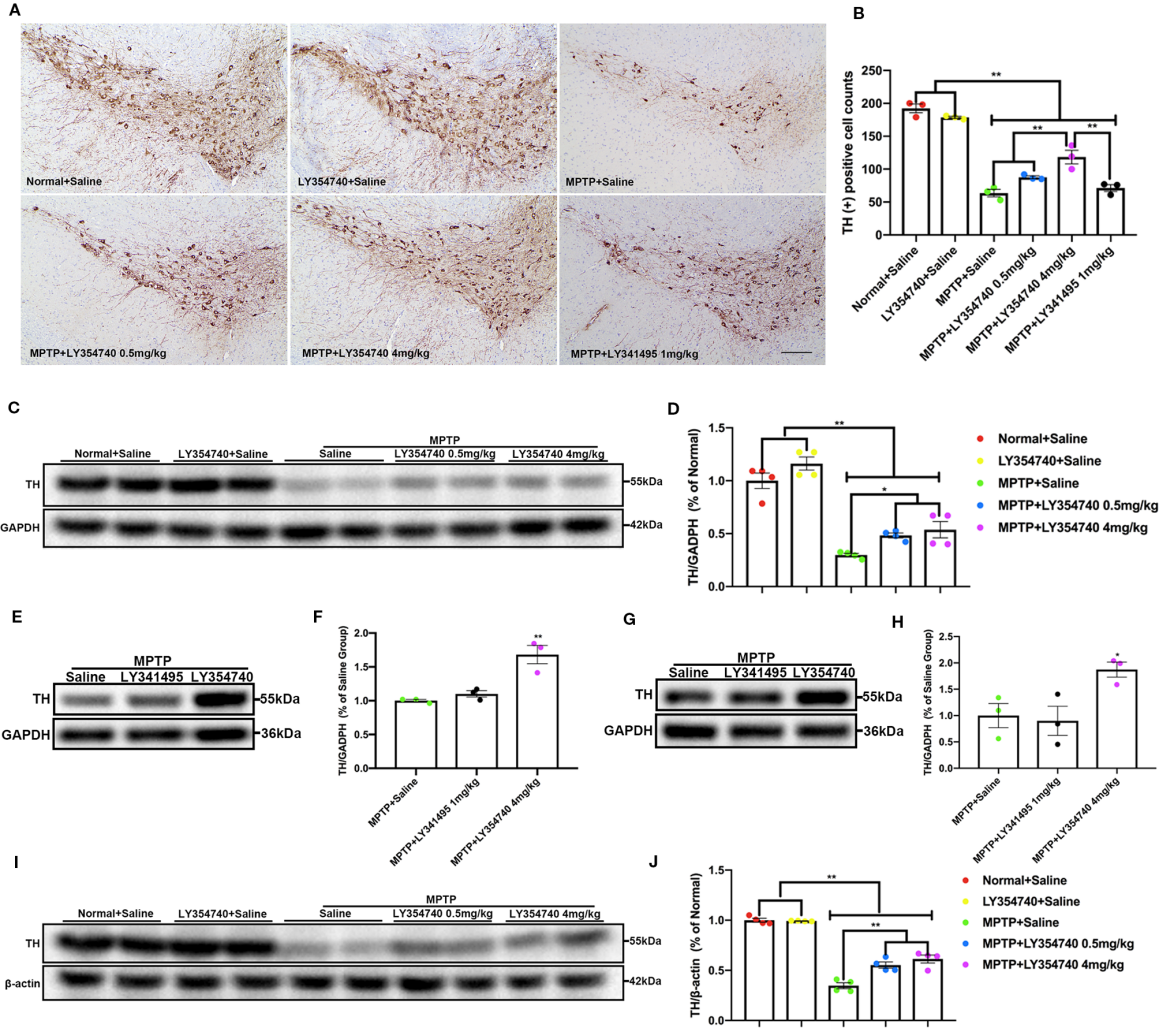
The effect of ligands of mGlu_2/3_ receptors on expression of tyrosine hydroxylase (TH) in substantia nigra (SN) and striatum in acute 1-methyl-4-phenyl-1,2,3,6-tetrahydropyridine (MPTP)-induced Parkinson’s disease (PD) mice models. **(A)** Immunohistochemical images of tyrosine hydroxylase (TH) in SN of each group. Scale bar = 100 μm. **(B)** Analysis of differences in TH positive cell counts in SNc from each group. ^**^ *p* < 0.01. *N* = 3. Error bars represent SEM. Western blot analysis showed levels and relative quantification of TH in acute-MPTP mice in SN **(C–F)** of each group. ^*^ *p* < 0.05, ^**^ *p* < 0.01, N = 4 in **(D)**; ^**^ *p* < 0.01 vs. other groups; N = 3 in **(F)**. Western blot analysis showed the levels and relative quantification of expression of TH in striatum **(G–J)** of acute-MPTP mice treated with different doses of LY354740. ^*^ *p* < 0.05 vs. the other groups; ^**^ *p* < 0.01. *N* = 4 in **(J)**, N = 3 in **(H)**. Data are expressed as % of the normal + saline group in **(D, J)**. Data are expressed as % of the MPTP + saline group in **(F, H)**. Error bars represent SEM.

To explore whether the different extents of the dopaminergic lesions may affect the pharmacological effects of LY354740, we employed acute MPTP mice model, which may induce severe degenerative changes. We assessed the integrity of dopaminergic neurons in the SN and their terminal fibers in the striatum. We found that there were fewer TH positive neurons in MPTP-treated groups compared with the normal + saline group and LY354740 + saline group in the similar anatomic level sections of SN (p < 0.01; **Figures 6A, B**). MPTP + LY354740 (4 mg/kg) group expressed more TH positive cells than the other three MPTP-treated groups (all p < 0.01; **Figures 6A, B**). In addition, no significant difference was found between MPTP + saline group, MPTP+LY354740 (0.5 mg/kg) group and the MPTP + LY341495 group. We noticed that, LY354740 (0.5 and 4 mg/kg) produced a dose-dependent increase in the number of TH positive cells compared with the MPTP + saline group.

We also quantified the level of TH in SN (**Figures 6C–F**) and striatum (**Figures 6G–J**) *via* western blot analysis. All the mice treated with MPTP expressed lower TH compared with both normal + saline group and LY354740 + saline group (p < 0.01). In addition, MPTP + LY354740 (0.5 or 4 mg/kg) expressed higher TH than MPTP + saline group in both SN (p < 0.05) and striatum (p < 0.01). No significant difference was found between MPTP + LY341495 (1 mg/kg) group and MPTP + saline group in both SN and striatum. We conclude that pretreatment with LY354740 plays a protective role against acute-MPTP toxicity.

### mGlu_2/3_ Receptors Ligands Regulate Expression of Phosphorylation of Fyn/NMDAR, PLK2, and pS129 α-Synuclein in Acute MPTP Mice Models

**Figure 7:**
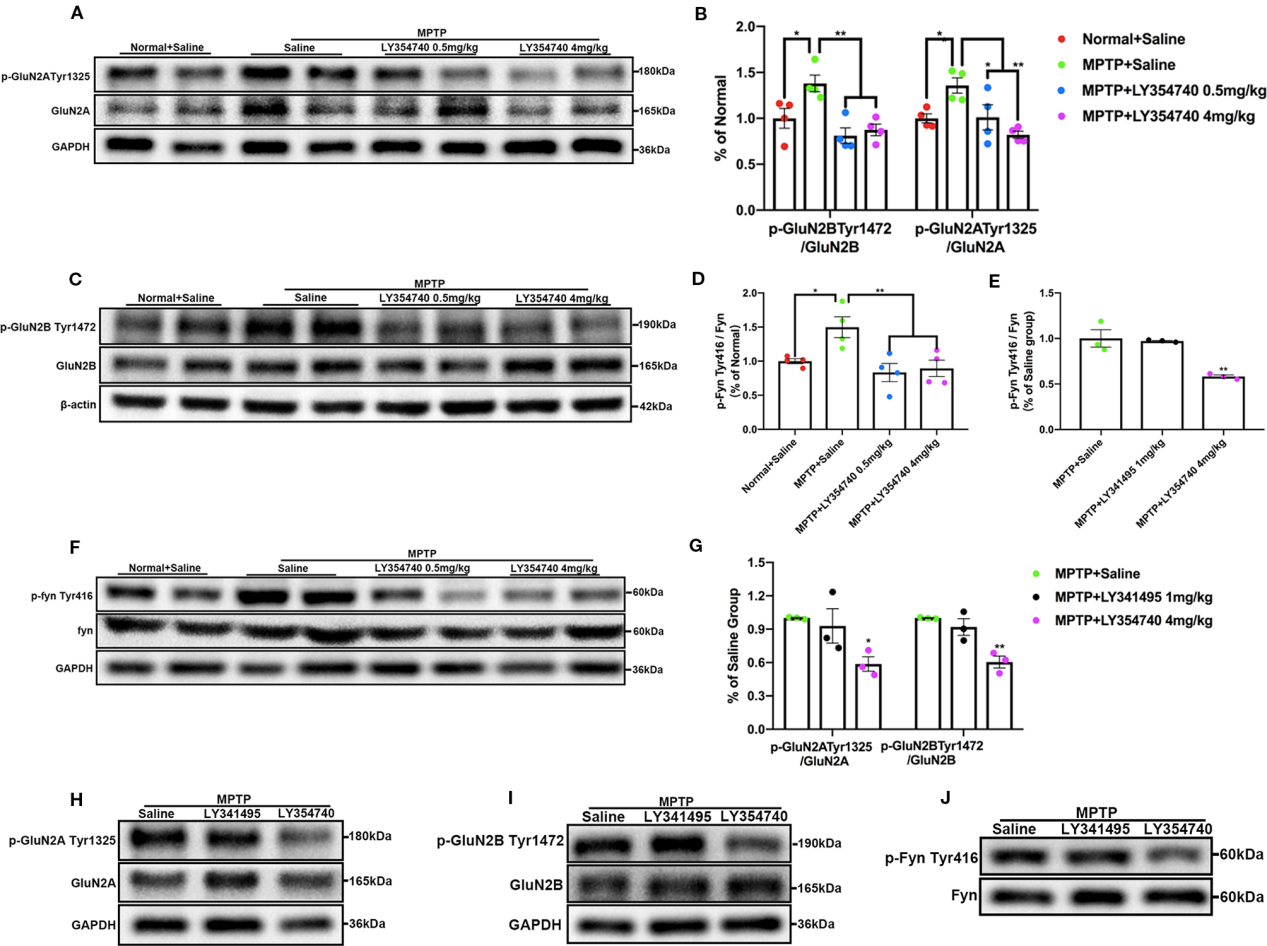
mGlu_2/3_ receptor ligands regulate expression of phosphorylation of Fyn/N-methyl-D-aspartate receptors (NMDAR) in acute 1-methyl-4-phenyl-1,2,3,6-tetrahydropyridine (MPTP) mice models. Immunoblots and relevant histograms show expression of phosphorylated NMDAR subunit GluN2A at Tyr1325 **(A, B),** GluN2B at Tyr1472 **(B, C),** Fyn at Tyr416 **(D, F)** in substantia nigra (SN) of acute MPTP-treated mice with different doses of LY354740. The band intensity of particular target proteins was normalized to GluN2A, GluN2B or Fyn levels. ^*^p < 0.05, ^**^p < 0.01. N = 4. Western blots represent the expression of phosphorylated NMDAR subunit GluN2A at Tyr1325 **(G, H),** GluN2B at Tyr1472 **(G, I)** and Fyn at Tyr416 **(E, J)** in acute MPTP-treated mice with saline, LY341495 or LY354740. The band intensity of particular target proteins was normalized to GluN2A, GluN2B or Fyn levels. ^*^p < 0.05, ^**^p < 0.01 vs. the other two groups. N = 3. Data in **(B, D)** were expressed as % of the normal + saline group, and data in **(E, G)** were expressed as % of the MPTP + saline group. Error bars represent SEM.

The results of western blot analysis of SN lysates showed that the ratio of p-GluN2A Tyr1325/GluN2A (**Figures 7A, B**), p-GluN2B Tyr1472/GluN2B (**Figures 7B, C**), and p-Fyn Tyr416/Fyn (**Figures 7D, F**) were much higher in acute MPTP + saline group compared with the normal + saline group (p < 0.05). Ratios of p-Fyn Tyr416/Fyn (p < 0.01) and p-GluN2B Tyr1472/GluN2B (p < 0.01) were down-regulated when the acute MPTP mice were pretreated with LY354740 (0.5 or 4 mg/kg) compared with the MPTP + saline group. Ratio of p-GluN2A Tyr1325/GluN2A was also decreased in the MPTP + LY354740 (0.5 mg/kg) group (p < 0.05) and MPTP + LY354740 (4 mg/kg) group (p < 0.01) compared with the MPTP + saline group. No significant difference was found between the normal + saline group and the MPTP + LY354740 groups. No obvious difference was found between the MPTP + saline group and the MPTP + LY341495 group in all ratios mentioned above (**Figures 7E, G–J**). Hence, the acute MPTP treatment might result in overexpression of p-Fyn Tyr416, p-GluN2A Tyr1325, and p-GluN2B Tyr1472, while LY354740 could down-regulated the abnormal expressions. All the above data revealed that LY354740 might have a neuroprotective effect associated with down-regulating abnormal phosphorylated Fyn and NMDAR subunits.

**Figure 8:**
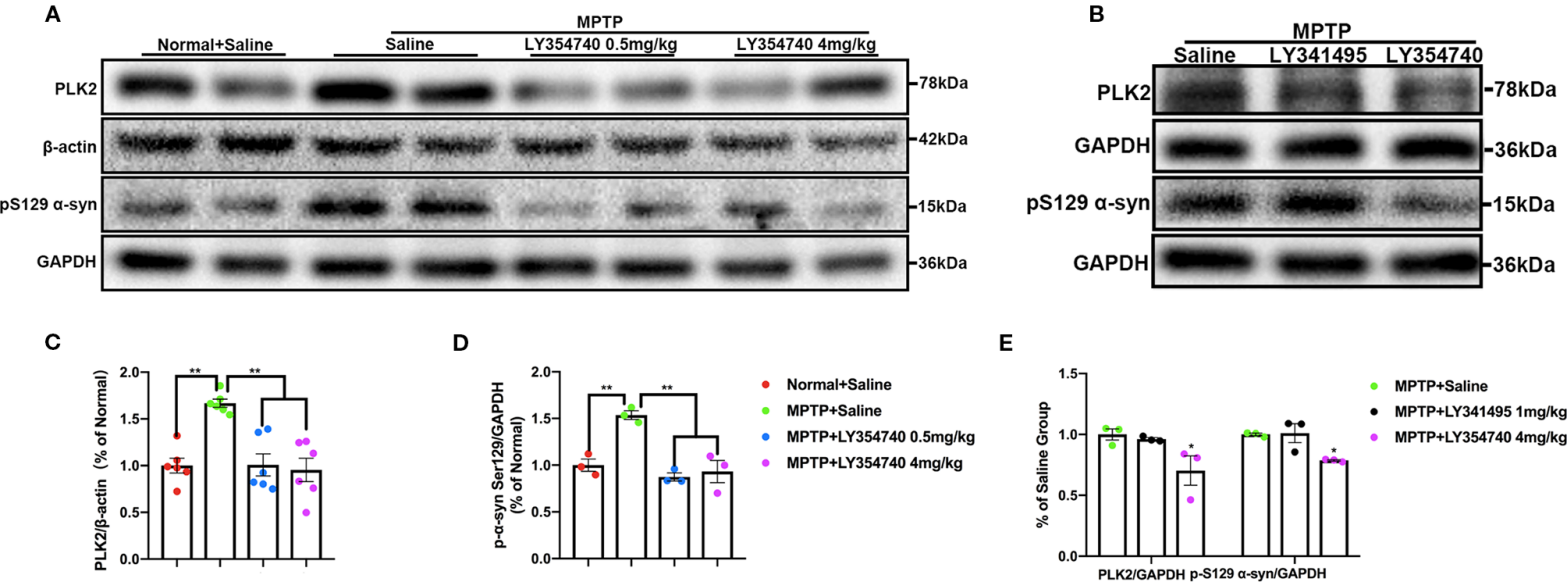
The effect of ligands of mGlu_2/3_ receptors on expression of polo-like kinase 2 (PLK2) and phosphorylated α-synuclein at Ser129 in substantia nigra (SN) of acute 1-methyl-4-phenyl-1,2,3,6-tetrahydropyridine (MPTP)-induced Parkinson’s Disease (PD) mice model. Immunoblots **(A)** and relevant histograms **(C, D)** represent expression of PLK2 and phosphorylated α-synuclein at Ser129 in SN of each group. Band intensity of particular target proteins were normalized to β-actin or glyceraldehyde 3-phosphate dehydrogenase (GAPDH) level. ^**^p < 0.01. N = 6 in **(C)**; N = 3 in **(D)**. Data were expressed as % of the normal + saline group and shown as the mean ± SEM. **(B, E)** Western blots represent expression of PLK2 and pS129 α-synuclein in SN of acute MPTP-treated mice with different drug intervention. ^*^p < 0.05 compared with the other two groups. N = 3. Data were expressed as % of the MPTP + saline group and shown as the mean ± SEM.

We examined the level of PLK2 and pS129 α-synuclein in the SN and found that compared with the normal + saline group, the levels of PLK2 and pS129 α-synuclein were significantly higher in the acute MPTP + saline group (p < 0.01; **Figures 8A, C, D**). Meanwhile, MPTP-mice treated with LY354740 (0.5 mg/kg or 4 mg/kg) evidently down-regulated the abnormal expression of PLK2 and pS129 α-synuclein compared with the MPTP-saline group (p < 0.01; **Figures 8A, C, D**). No significant difference was found between the MPTP-saline group and the MPTP+LY341495 group (**Figures 8B, E**). These results suggested that LY354740 might reduce PD-related pathology in acute MPTP-induced PD mice models by inhibiting expression of PLK2.

## Discussion

Excessive glutamate has been implicated as an excitotoxic agent in PD. Excitatory neurons of the subthalamic nucleus send glutamatergic projections to dopaminergic neurons of the substantia nigra pars compacta (SNc), which may contribute to the excitotoxic damage to SNc neurons in PD models (Conn et al., 2005; Duty, 2012; Sebastianutto and Cenci, 2018). Consideration of mitochondrial respiration deficits and subsequent cellular oxidative stress caused by MPTP, dopaminergic neurons of SNc may not be able to cope with elevation of intracellular Ca^2+^ followed by extracellular glutamatergic stimulation (Jiang and Dickson, 2018). Early studies have demonstrated neuroprotective effects in MPTP models with several kinds of mGlu_2/3_ receptors agonists including DCG-IV and LY379268 (Matarredona et al., 2001; Battaglia et al., 2003). Whether LY354740 provides an opportunity to slow down the degeneration of the dopaminergic system as LY379268 and DCG-IV is unknown.

LY354740 penetrates into the brain, allowing its central effects to be studied following systemic administration (Lilly, 2003). LY354740 exerts its effects in a number of animal models of brain pathologies, including schizophrenia, epilepsy, drug withdrawal, neurodegenerative diseases, ischemia/traumatic brain injury, panic disorder, anxiety (Konieczny et al., 1998; Kłodzińska et al., 1999; Schoepp et al., 1999). The published phase I/II clinical trials demonstrated its anxiolytic and/or antipanic effects with no adverse effects reported so far (such as sedation, ataxia and abuse liability) (Grillon et al., 2003; Schoepp et al., 2003; Dunayevich et al., 2008). These data above suggest that LY354740 might become a clinically available drug to slow down the degeneration of the dopaminergic system caused by glutamate overactivity.

In our experiments, LY354740 dose-dependently reduced dopaminergic neurons loss in both acute and sub-acute MPTP mice models, while LY341495 (1 mg/kg, i.p.) aggravated neurodegeneration caused by MPTP in sub-acute MPTP mice models. Neuronal degeneration also reflected in motor function and monoamines and glutamate content. LY341495 amplified the reduction in DA level, up-regulated expression of glutamate and aggravated the performances in the rotarod test in sub-acute MPTP mice. LY354740 did not significantly improve locomotor function, influence dopamine and glutamate contents in sub-acute mice models, maybe because the lesions were not as severe enough to indicate apparent differences (Zhang et al., 2017). Meanwhile, LY354740 down-regulated the level of glutamate and improved locomotor function in acute-MPTP mice. Interestingly, although LY354740 did not significantly alleviate DA loss in acute MPTP mice, LY354740 improved locomotor function obviously. This might because the beneficial effects of locomotor observed in PD models after systemic administration of mGlu2/3 agonists could derive from the reduction of both corticostriatal transmission and subthalamic nucleus hyperactivity (Rouse et al., 2000; Murray et al., 2002) and the content of dopamine in striatum might not influence its antiparkinsonian properties (Konieczny et al., 1998; Ali and Salter, 2001).

Postsynaptic glutamate receptors are only activated when glutamate binds to them from the outside. Thus, mechanisms which reduce extracellular concentration of glutamate are essential for protecting dopaminergic cells from excitatory amino acid toxicity. We found that chronic injection of LY354740 decreased extracellular glutamate concentration in both acute and sub-acute MPTP mice. Thirty minutes after LY354740 injection, extracellular concentrations of glutamate further decreased. However, although the dose of LY354740 used in sub-acute MPTP mice was much higher than that in acute MPTP mice, a greater change in glutamate concentration (before and after LY354740 injection) was found in the acute MPTP/LY354740 group. This might be related to astrocytes. Basal extracellular glutamate concentration was consistent with expression of astrocytes (Han et al., 2008). We demonstrated that mice pretreated with sub-acute MPTP/LY354740 contained less extracellular glutamate and astrocytes than mice pretreated with acute MPTP/LY354740. Thus, more astrocytes might be activated for more extracellular glutamate uptake in acute MPTP/LY354740 mice than that in sub-acute MPTP/LY354740 mice.

Our results with LY354740 support modulatory role of mGlu_2/3_ receptors to prevent excessive extracellular glutamate concentration *in vivo*. How dose LY354740 modulate extracellular glutamate concentration? Glutamate is stored presynaptically in glutamatergic nerve terminals where it can be released by a Ca^2+^-dependent manner (Fonnum, 1984). Specifically, mGlu_2/3_ receptors are expressed presynaptically on subthalamonigral glutamatergic terminals, far from the active zone of neurotransmitter release (Nicoletti et al., 2011). Activation of mGlu_2/3_ receptors produces inhibitory effects mediated by a decreased activity of voltage-gated Ca^2+^ channels (Pin and Duvoisin, 1995). The major function of presynaptic mGlu_2/3_ receptors is to act as auto-receptors to provide negative feedback on presynaptic glutamate release (Bradley et al., 2000; Niswender and Conn, 2010; Johnson et al., 2011). In addition, action of extracellular glutamate is terminated by uptake into the surrounding astrocytes *via* excitatory amino acid transporter 2 (EAAT2) (Kam and Nicoll, 2007). mGlu_3_ subtype also presents in glial cells, especially the astrocytes (Durand et al., 2013). Activation of mGlu_3_ receptor induces EAAT2 expression in astrocytes which contributes to enhance glutamate uptake and modulate extracellular glutamate at synapses (Yao et al., 2005; Mudo et al., 2007). Astrocytes also respond to neurotransmitters with an increase of intracellular Ca^2+^ and then result in the release of gliotransmitters, such as glutamate. In rat cortical neuronal cultures, LY354740 protects neurons from NMDA toxicity through activation of presynaptic mGlu_2/3_ receptors which reduce glutamate release, while this neuroprotective effect is enhanced by the presence of astrocytes (Kingston et al., 1999). The results of Kingston’s group indicated that activation of mGlu_3_ receptor might not play a role in release of glutamate from astrocytes and the protective mechanism of LY354740 might quite complex involving both neurons and astrocytes. Indeed, due to the complex interplay between astrocytes and neurons *in vivo*, it does not enable us to ascertain whether modulations of glutamate concentration achieved were due to inhibition of presynaptic glutamate release, reduction of gliotransmitter release, enhancement of glutamate uptake by astrocytes or the combination of these targets. In fact, since the pathology of PD is multifactorial, therapies aimed at modulating multiple pathways (including neurons and astrocytes) may be more effective than those directed at a single target. Future experiments will be fundamental to further define the role of mGlu_3_ receptor in gliotransmitter and the complex interplay between astrocytes and neurons occurring in neurological disease.

High concentrations of glutamate ultimately lead to overactivity of postsynaptic NMDA receptors. It is now unanimously accepted that NMDARs play a major role in excitotoxic cell death. Functional NMDARs contain obligatory GluN1 and modulatory GluN2 subunits. Studies showed that NMDARs activation, including synaptic currents and number of NMDARs on the cellular surface, are governed by tyrosine phosphorylation and dephosphorylation of GluN2 subunits, especially GluN2A Tyr1325 and GluN2B Tyr1472 (Ali and Salter, 2001; Salter and Kalia, 2004; Trepanier et al., 2012). Fyn, a member of the Src kinase family, is colocalized with NMDA receptors at postsynaptic density (Tezuka et al., 2002). Tyr416 phosphorylation has been widely used as an activation-associated marker of Fyn. Activated Fyn was shown to phosphorylate both GluN2A and GluN2B at tyrosine sites (Suzuki and Okamuranoji, 1995), resulting in increased membranal retention and enhanced channel activity (Salter and Kalia, 2004). Studies have demonstrated that, in Fyn-depleted mice, redistribution of NMDA receptors was drastically reduced (Dunah, 2004). In our study, we found that compared with normal mice, MPTP-treated mice abnormally up-regulated expression of p-Fyn Tyr416/p-GluN2B Tyr1472/p-GluN2A Tyr1325. Both acute and sub-acute MPTP mice pre-treated with LY354740 dose-dependently reduced all abnormal expressions above. Mice pretreated with LY341495 exhibited far higher expression of p-Fyn Tyr416/p-GluN2B Tyr1472 in the sub-acute MPTP model only, compared with MPTP mice treated with vehicle. In acute MPTP mice models, insults were stronger than those in sub-acute MPTP-treated mice, and the postsynaptic glutamate receptor might be saturated by endogenous glutamate. This might explain why LY341495 showed no significant activity under these conditions. These results indicated that mGlu_2/3_ receptors ligands might exert effects on regulating the activation of Fyn/NMDARs.

It is well known that α-synuclein is a primary factor in the pathogenesis of PD. Nearly 90% of α-synuclein in Lewy bodies is phosphorylated on Ser129 (Samuel et al., 2016). PLK2 was regarded as an important phosphorylation-related kinase in mediating α-synuclein phosphorylation at Ser129, both *in vivo* and *in vitro* (Inglis et al., 2009; Waxman and Giasson, 2011; Basso et al., 2013; Bergeron et al., 2014). In cultured hippocampal neurons, glutamate stimulation could elevate expression of PLK2. Furthermore, this elevation could be blocked by the NMDA receptor antagonist (Wu et al., 2007). Identical with protein level, transcription of the PLK2 gene is responsive to long-term potentiation and neuronal increased oxidative stress (Kauselmann et al., 1999; Wang et al., 2019). These results demonstrated that PLK2 might play a role in glutamate-induced excitotoxicity and provide a potential link between excitotoxic responses and Lewy pathology. In our study, we found that, both acute and sub-acute MPTP mice pre-treated with LY354740, dose-dependently reduced expression of PLK2/pS129 α-synuclein. We proved that LY354740 could decrease extracellular glutamate in MPTP mice. These changes might be the mechanisms that lead to down-regulated expression of PLK2/pS129 α-synuclein. Simultaneously, LY341495 up-regulated the expression of PLK2/pS129 α-synuclein in sub-acute MPTP mice models, which further demonstrated the role of mGlu_2/3_ receptors in influencing the pathogenesis of PD.

Notably, positive allosteric modulators (PAM) continue to be an active field of research due to its fewer adverse effects, higher selectivity and greater structural diversity compared with orthosteric ligands (Engers and Lindsley, 2013). Two mGlu_2_ receptor PAMs have already advanced into schizophrenia and smoking cessation clinical trials (Trabanco et al., 2019). It was shown that activation of mGlu_3_ receptor is required for the neuroprotective effects of mGlu_2/3_ receptors agonists toward NMDA neurotoxicity in mixed cultures of astrocytes and neurons, whereas enhancers of mGlu_2_ receptor may amplify neuronal death (Corti et al., 2007). As of late, no selective mGlu_3_ receptor PAMs have been reported. Fortunately, Dhanya et al. reported that they developed mGlu_2/3_ receptors PAM (Dhanya et al., 2014). The protective and therapeutic potential of mGlu_2/3_ receptors and mGlu_3_ receptor PAM in PD models await further investigation.

In conclusion, the current study further confirmed that mGlu_2/3_ receptors play an important role in MPTP-treated mice. While LY341495 aggravated toxin-induced midbrain dopaminergic neuron degeneration in sub-acute MPTP-induced PD mice, LY354740 could protect dopaminergic cells in MPTP-treated mice, which might be related to the decreased extracellular glutamate concentration and down-regulated expression of p-fyn/NMDA and PLK2/pS129 α-synuclein in SN of MPTP-treated mice.

## Data Availability Statement

The raw data supporting the conclusions of this article will be made available by the authors, without undue reservation, to any qualified researcher.

## Ethics Statement

The animal study was reviewed and approved by The Institutional Animal Care and Use Committee at Tongji Medical College, Huazhong University of Science and Technology.

## Author Contributions

YT, YX, and XC contributed conception and design of the study. YT, YX, CC, CZ, WZ, JiW, XZ, JiaW, and XiaomeiY performed the experiments. SN performed the statistical analysis. YT and XiaomanY wrote the first draft of the manuscript. All authors contributed to manuscript revision, read and approved the submitted version.

## Funding

This work was supported by the National Natural Science Foundation of China (81974200, 81171193, 81671108 and 81873734) and the National Key R&D Program of China (2017YFC1310300).

## Conflict of Interest

The authors declare that the research was conducted in the absence of any commercial or financial relationships that could be construed as a potential conflict of interest.

## Abbreviations

PD, Parkinson’s disease; mGlu_2/3_, Group II metabotropic glutamate receptor; MPTP, 1-methyl-4-phenyl-1,2,3,6-tetrahydropyridine; GluN2A/2B: N-methyl-D-aspartate receptor subunit 2A/2B; PLK2, polo-like kinase 2; SNc, substantia nigra pars compacta; pS129 α-synuclein, phosphorylated α-synuclein at serine129.
