# Supplementary material for: LY354740 Reduces Extracellular Glutamate Concentration, Inhibits Phosphorylation of Fyn/NMDARs, and Expression of PLK2/pS129 α-Synuclein in Mice Treated With Acute or Sub-Acute MPTP
Source: Front Pharmacol. 2020 Feb 28;11:183. doi: 10.3389/fphar.2020.00183 (PMC7059821; doi:10.3389/fphar.2020.00183)

Sub-acute MPTP treatment

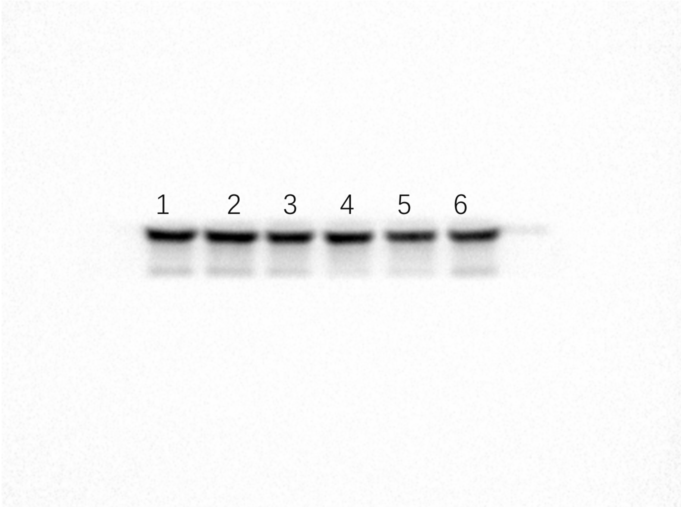

fig3C β-actin

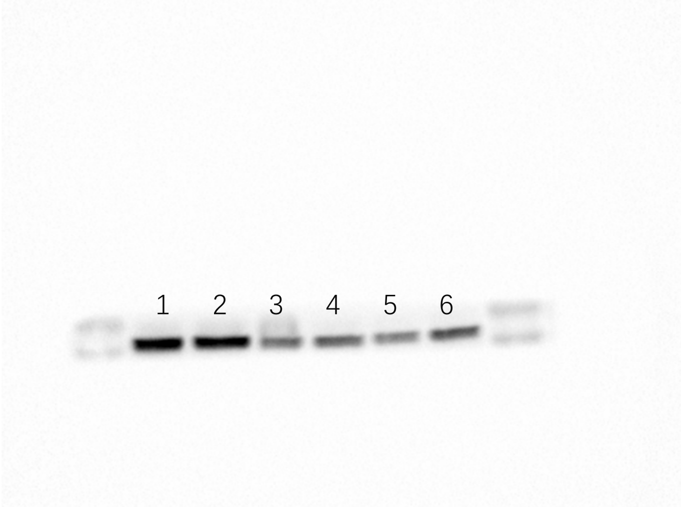

fig3C TH

1-Normal + Saline group
2-LY354740 + Saline group
3-MPTP + Saline group
4-MPTP + LY354740 0.1 mg/kg group
5- MPTP + LY354740 1 mg/kg group
6- MPTP + LY354740 10 mg/kg group

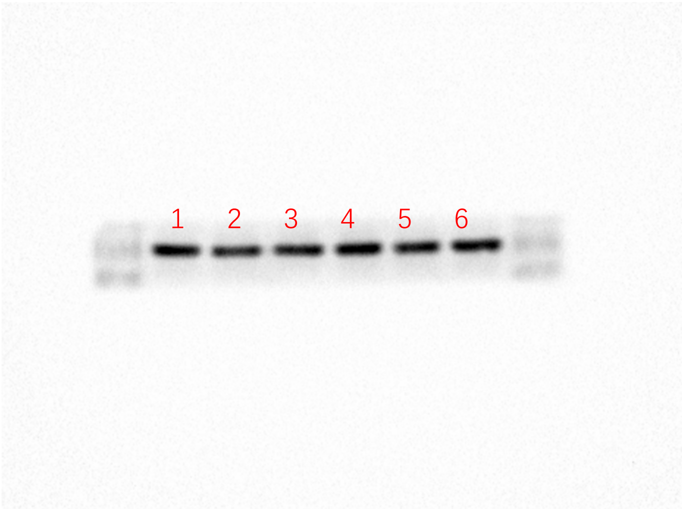

fig3H GAPDH

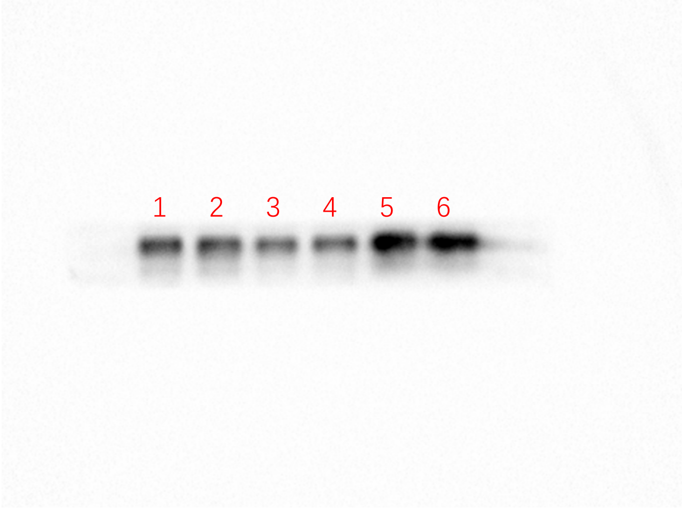

fig3H TH

1, 2-MPTP + Saline group

3, 4-MPTP + LY341495 1 mg/kg group

5, 6- MPTP + LY354740 10 mg/kg group

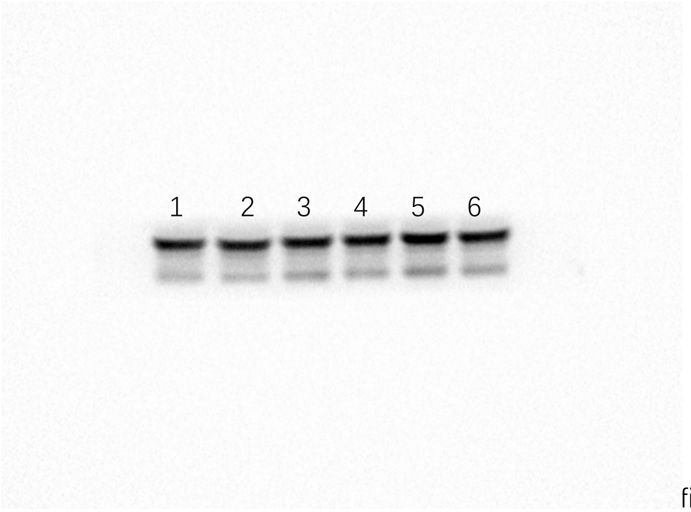

fig3D β-actin

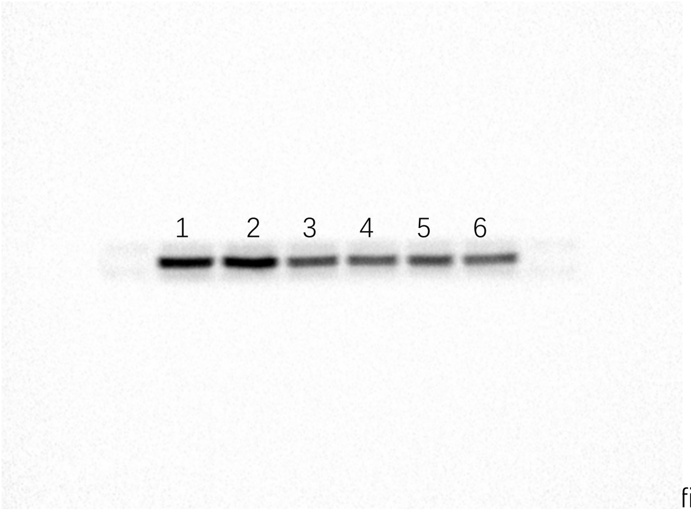

fig3D TH

1-Normal + Saline group
2-LY354740 + Saline group
3-MPTP + Saline group
4-MPTP + LY354740 0.1 mg/kg group
5- MPTP + LY354740 1 mg/kg group
6- MPTP + LY354740 10 mg/kg group

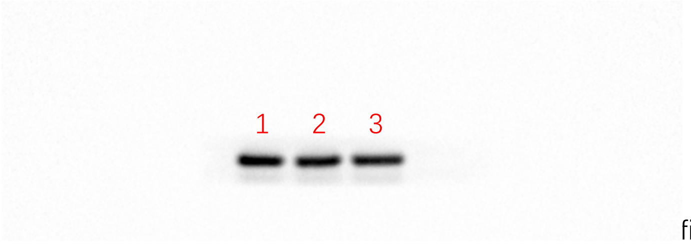

fig3I GAPDH

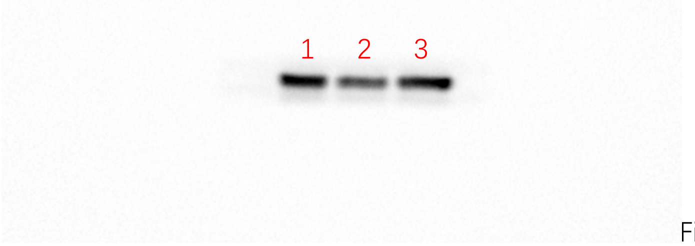

Fig3I TH

1-MPTP + Saline group

2-MPTP + LY341495 1 mg/kg group

3- MPTP + LY354740 10 mg/kg group

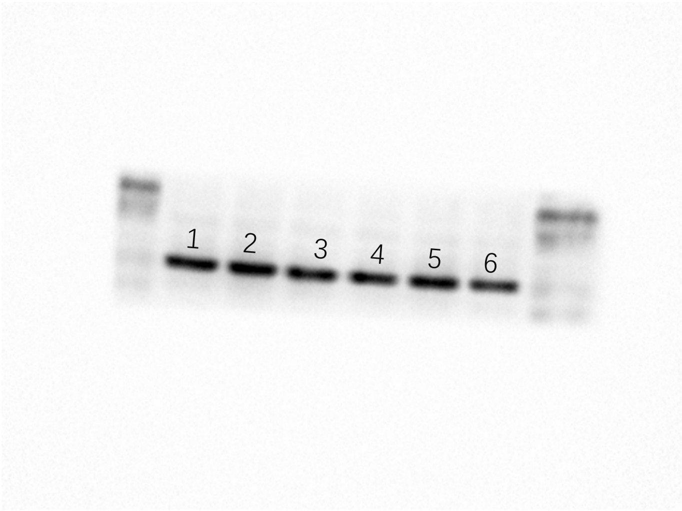

fig4A GAPDH

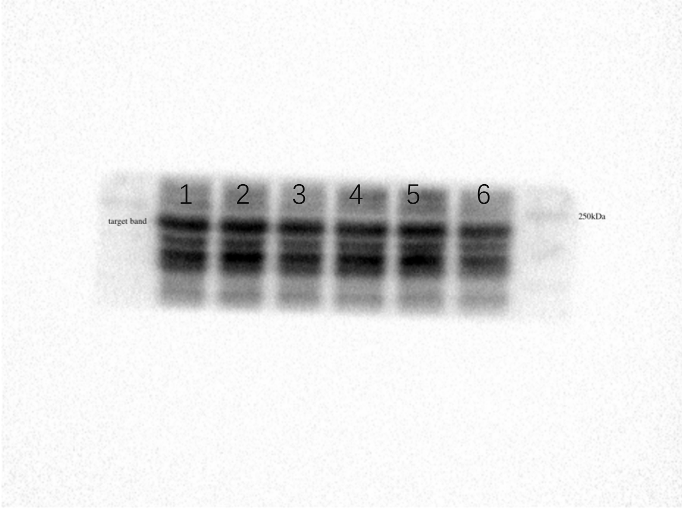

Fig4A GluN2A

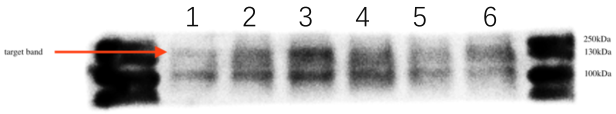

Fig4A p-GluN2A

1-Normal + Saline group；2-LY354740 + Saline group

3-MPTP + Saline group

4-MPTP + LY354740 0.1 mg/kg group

5- MPTP + LY354740 1 mg/kg group

6- MPTP + LY354740 10 mg/kg group

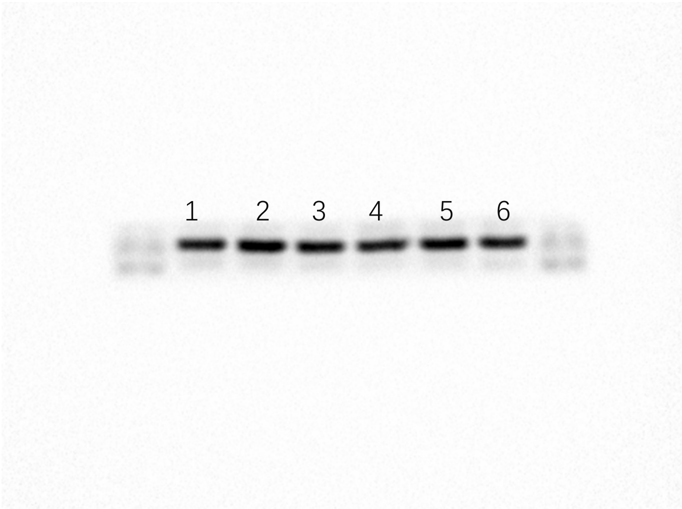

Fig4C GAPDH

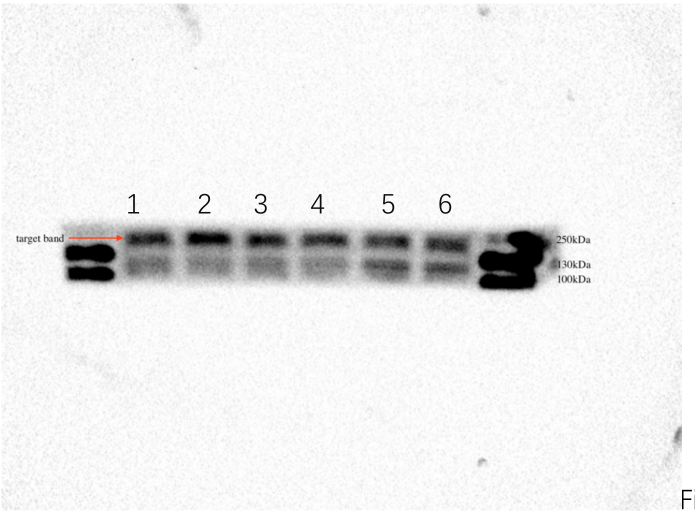

Fig4C GluN2B

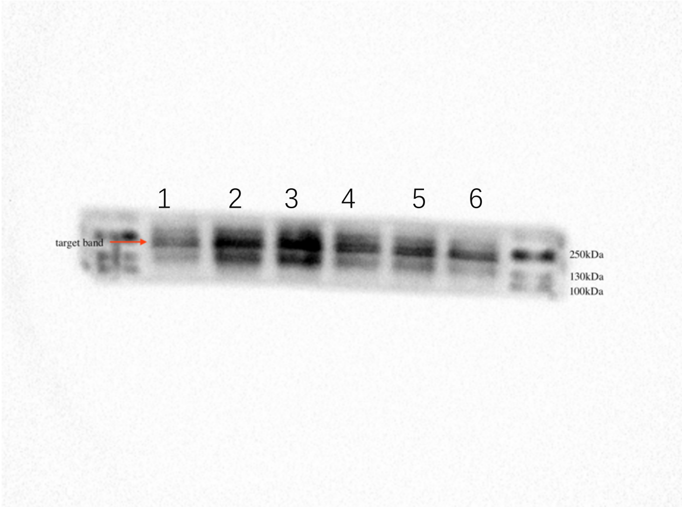

Fig4C p-GluN2B

1-Normal + Saline group；2-LY354740 + Saline group

3-MPTP + Saline group

4-MPTP + LY354740 0.1 mg/kg group

5- MPTP + LY354740 1 mg/kg group

6- MPTP + LY354740 10 mg/kg group

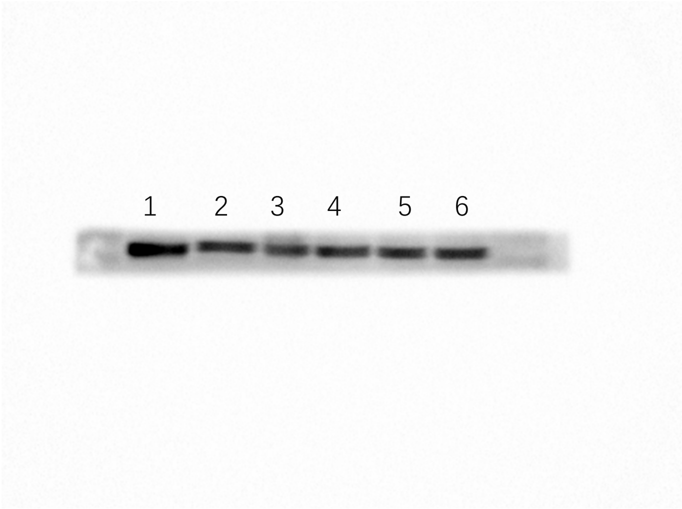

Fig4E fyn

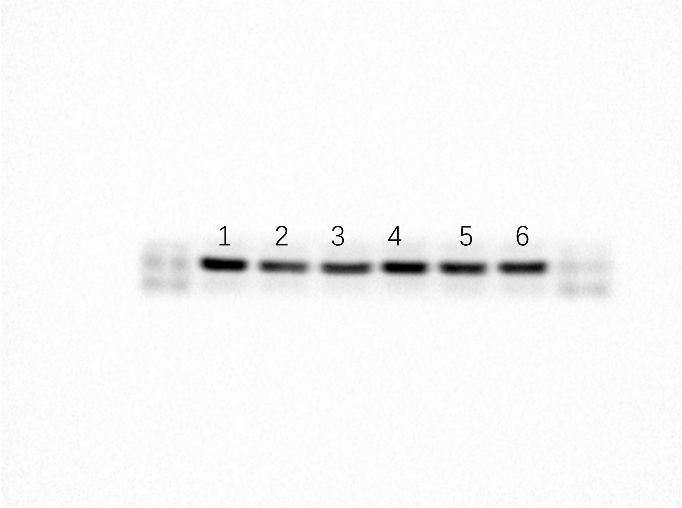

Fig4E GAPDH

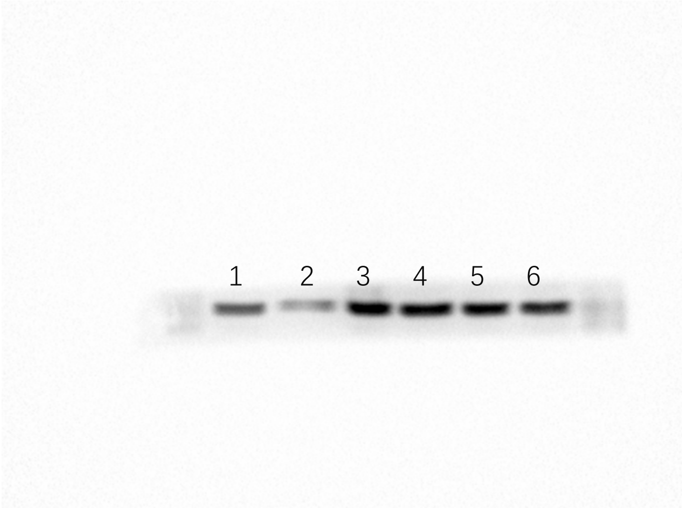

Fig4E p-fyn

1-Normal + Saline group；2-LY354740 + Saline group

3-MPTP + Saline group

4-MPTP + LY354740 0.1 mg/kg group

5- MPTP + LY354740 1 mg/kg group

6- MPTP + LY354740 10 mg/kg group

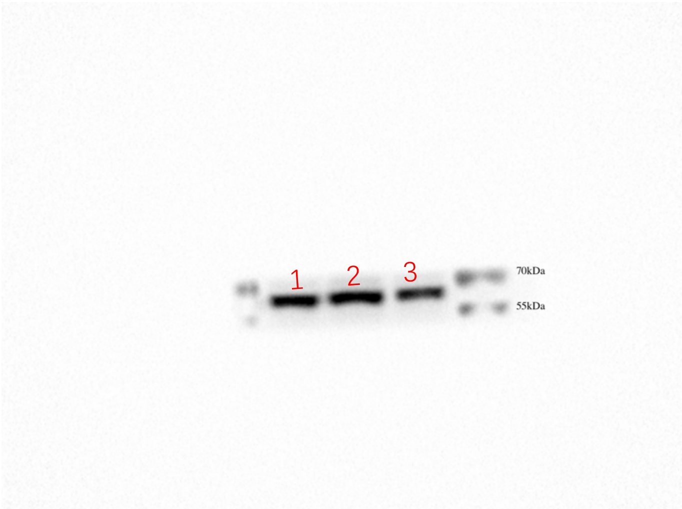

Fig4G fyn

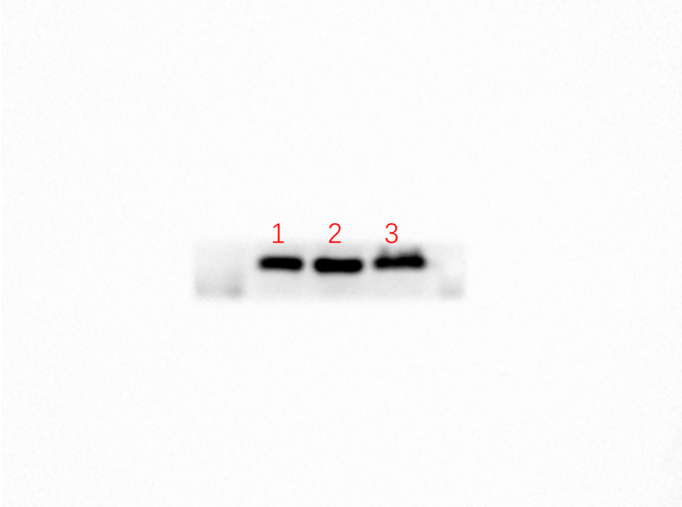

Fig4G GAPDH

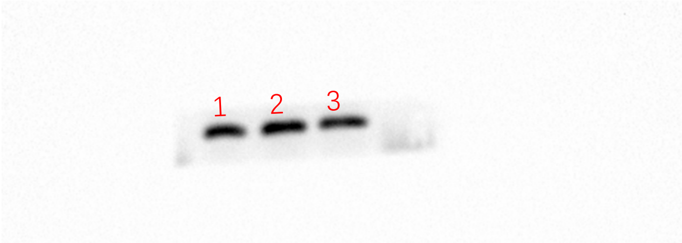

Fig4G GluN2A

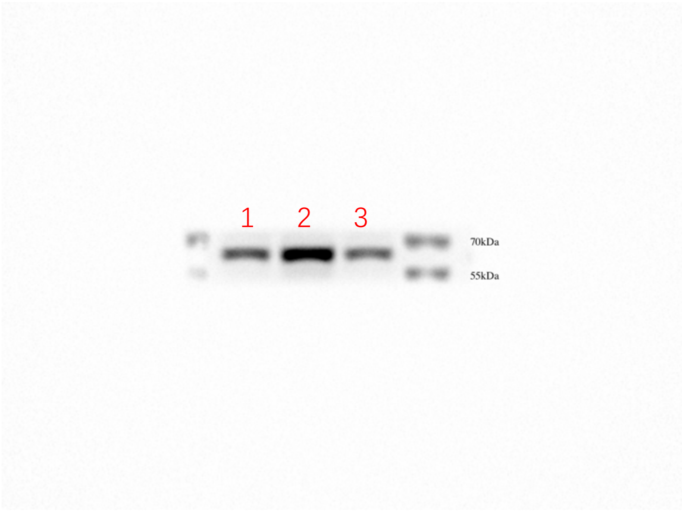

Fig4G p-fyn

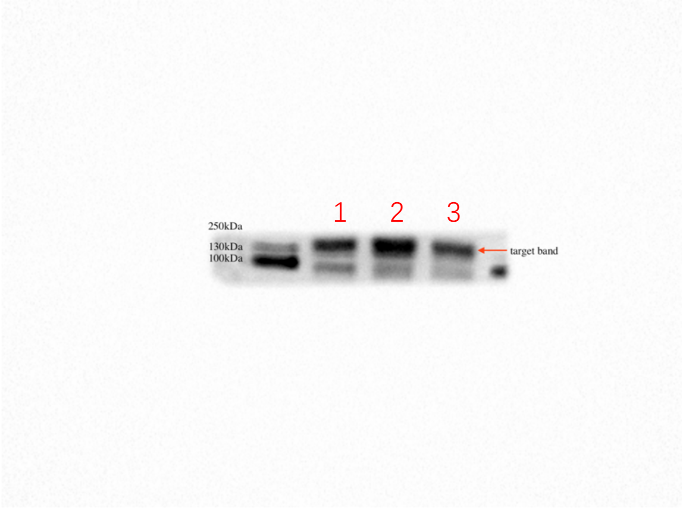

Fig4G p-GluN2A

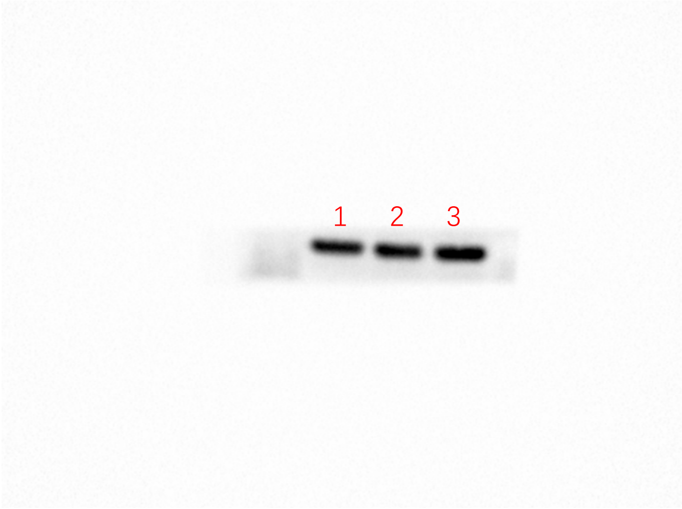

Fig4I GAPDH

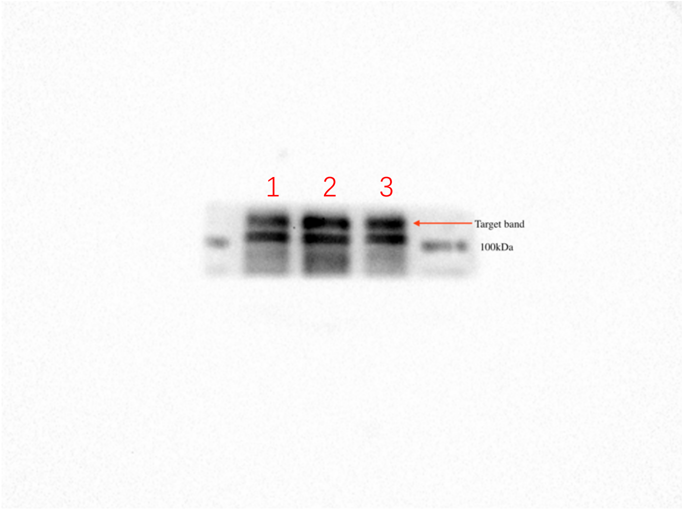

Fig4I GluN2B

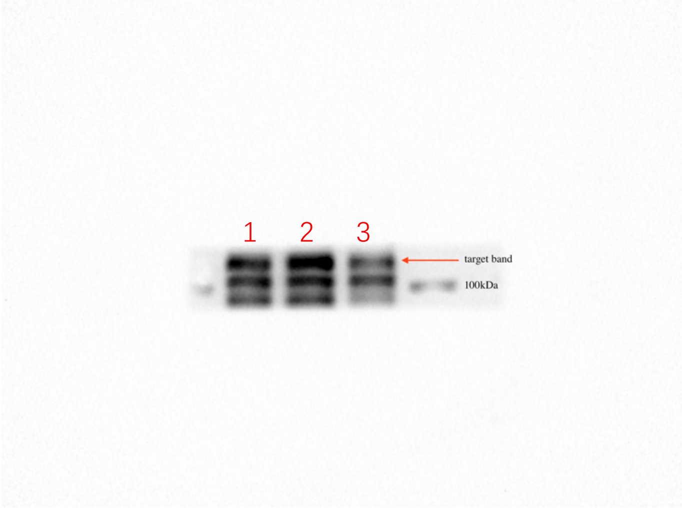

Fig4I p-GluN2B

1-MPTP + Saline group

2-MPTP + LY341495 1 mg/kg group

3- MPTP + LY354740 10 mg/kg group

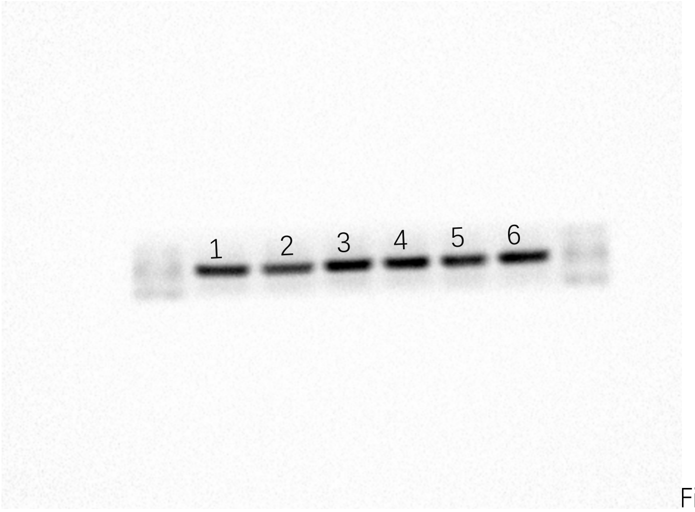

Fig5A GAPDH

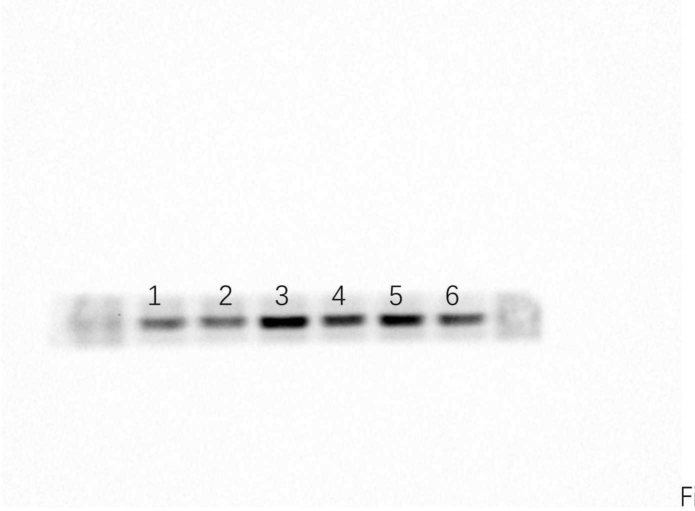

Fig5A PLK2

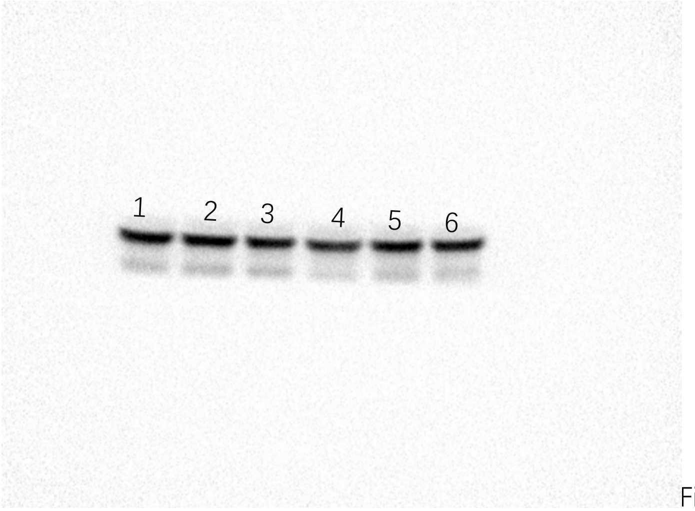

Fig5B actin

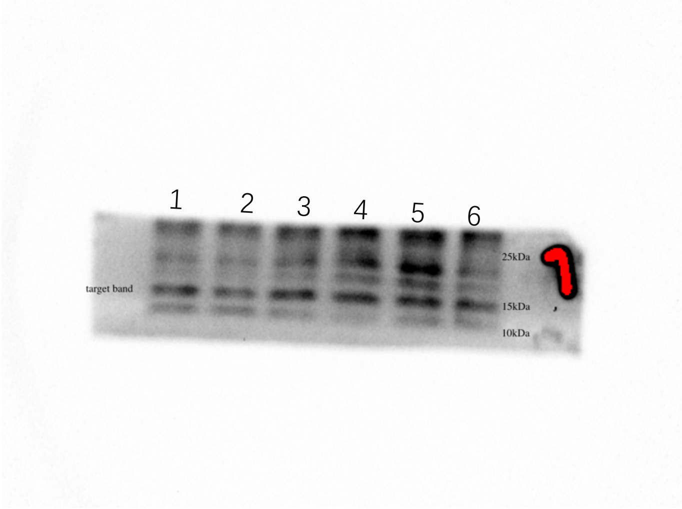

Fig5B p-syn

1-Normal + Saline group；2-LY354740 + Saline group

3-MPTP + Saline group

4-MPTP + LY354740 0.1 mg/kg group

5- MPTP + LY354740 1 mg/kg group

6- MPTP + LY354740 10 mg/kg group

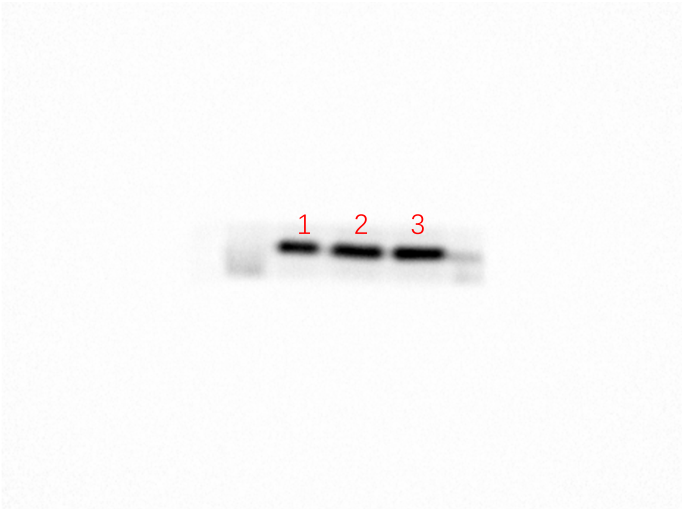

Fig5G GAPDH

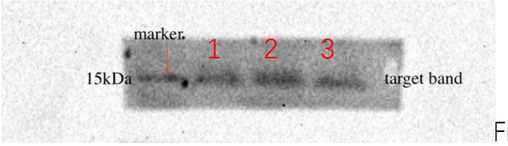

Fig5G p-syn

Fig5G PLK2

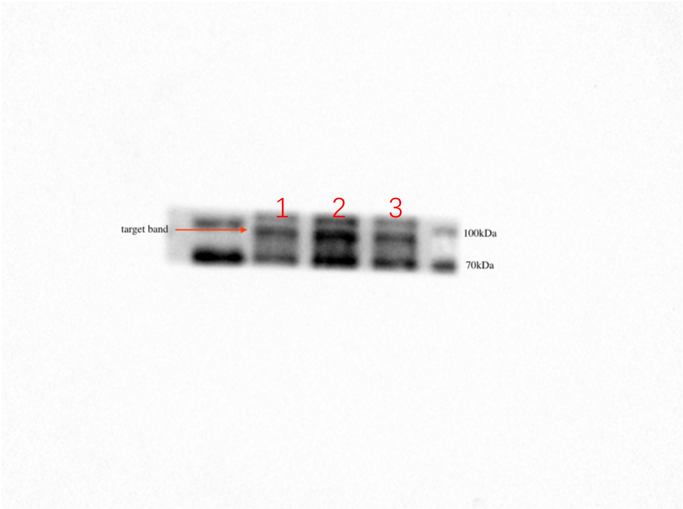

1-MPTP + Saline group

2-MPTP + LY341495 1 mg/kg group

3- MPTP + LY354740 10 mg/kg group

Acute MPTP treatment

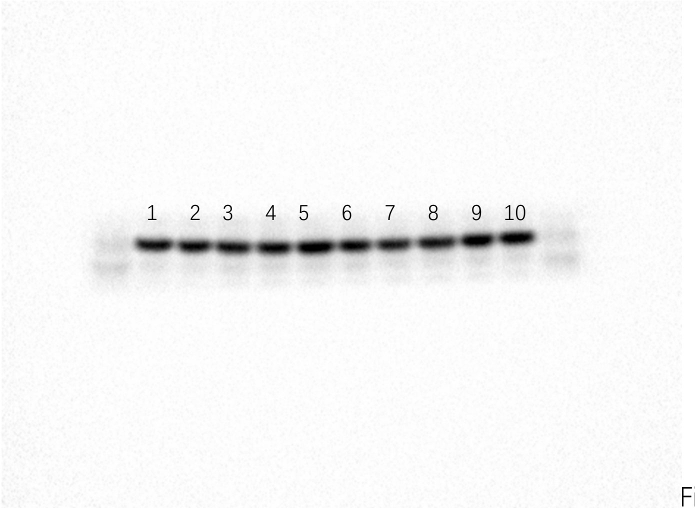

Fig6C GAPDH

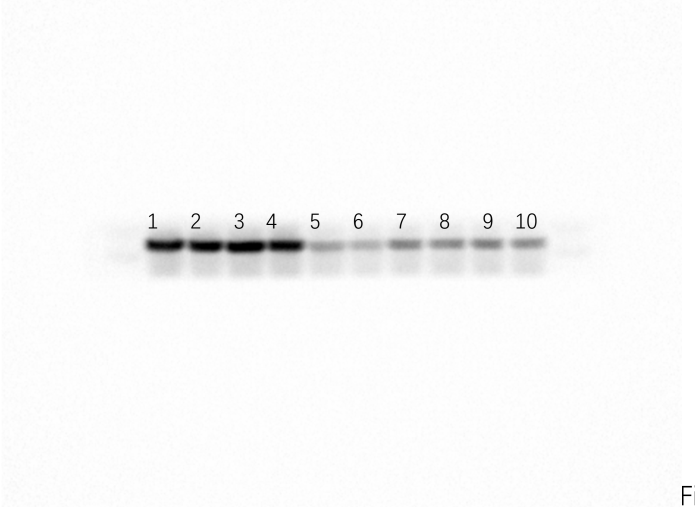

Fig6C TH

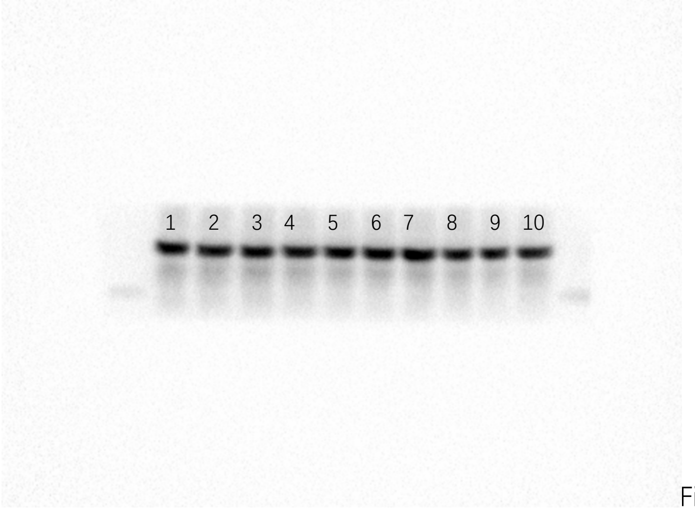

Fig6I Actin

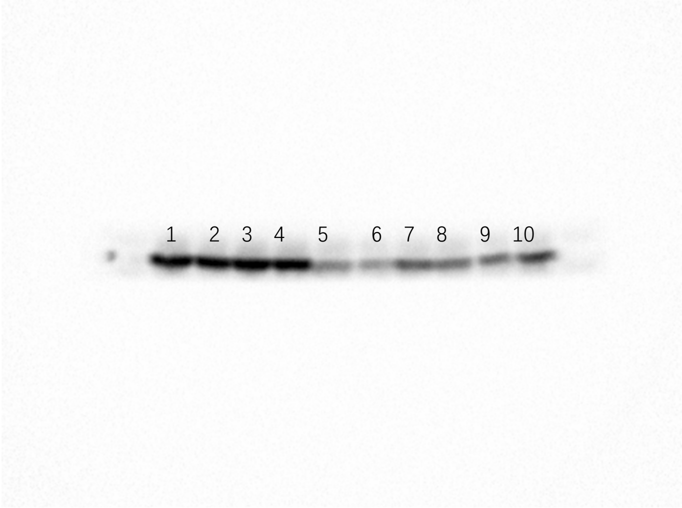

Fig6I TH

1, 2-Normal + Saline group

3, 4-LY354740 + Saline group

5, 6- MPTP + Saline group

7, 8- MPTP + LY354740 0.5 mg/kg group

9, 10- MPTP + LY354740 4 mg/kg group

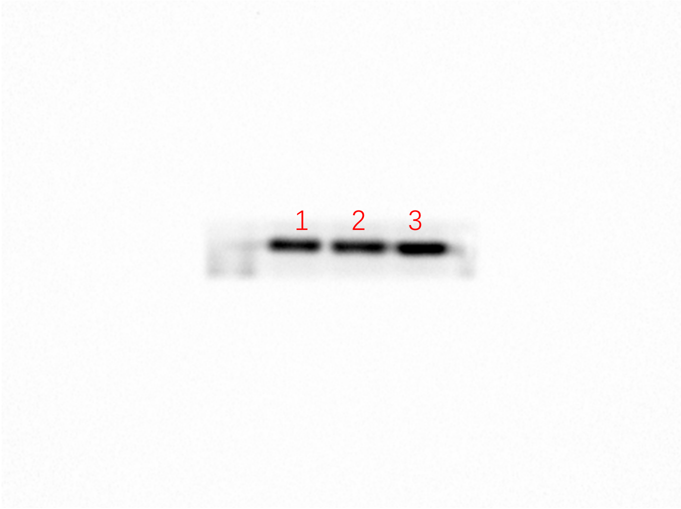

Fig6E GAPDH

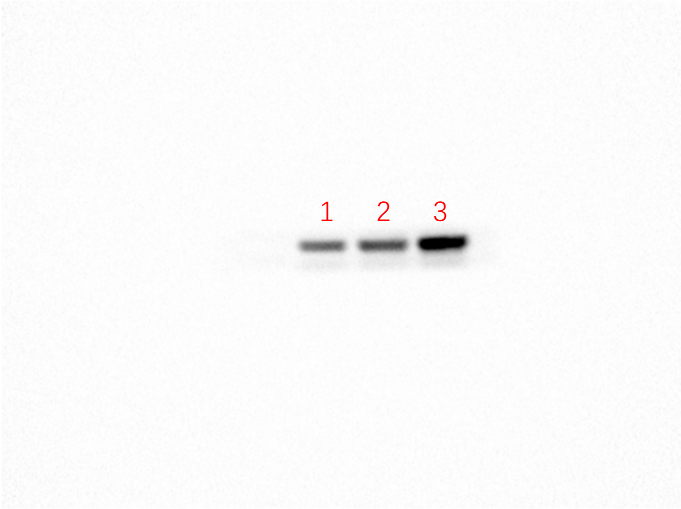

Fig6E TH

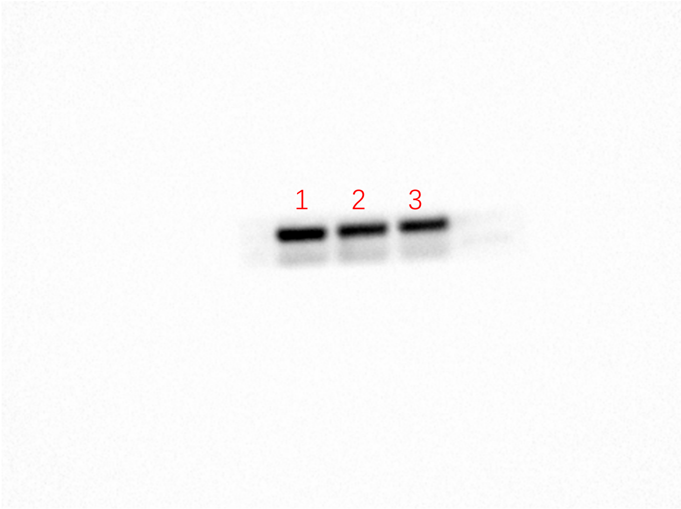

Fig6G GAPDH

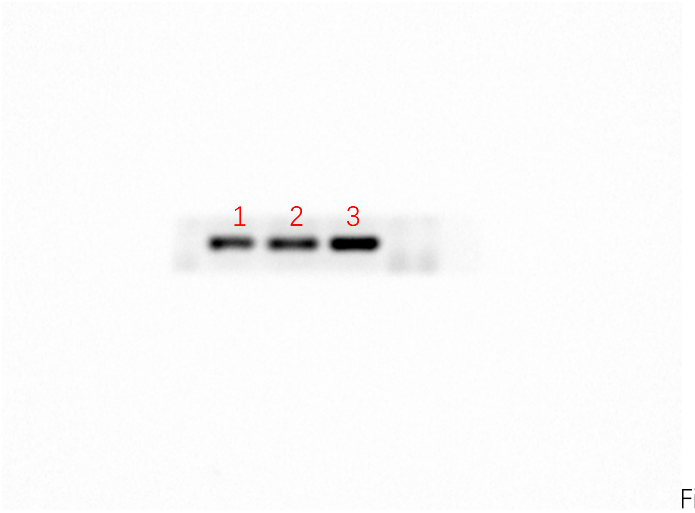

Fig6G TH

1-MPTP + Saline group

2-MPTP + LY341495 1 mg/kg group

3-MPTP + LY354740 4 mg/kg group

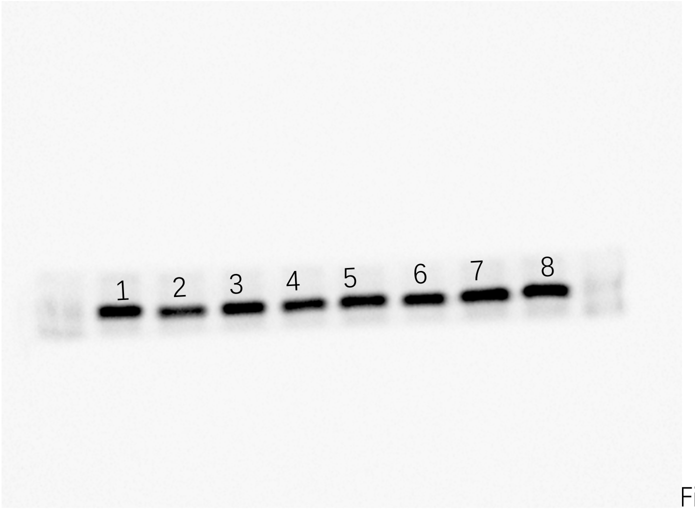

Fig7A GAPDH

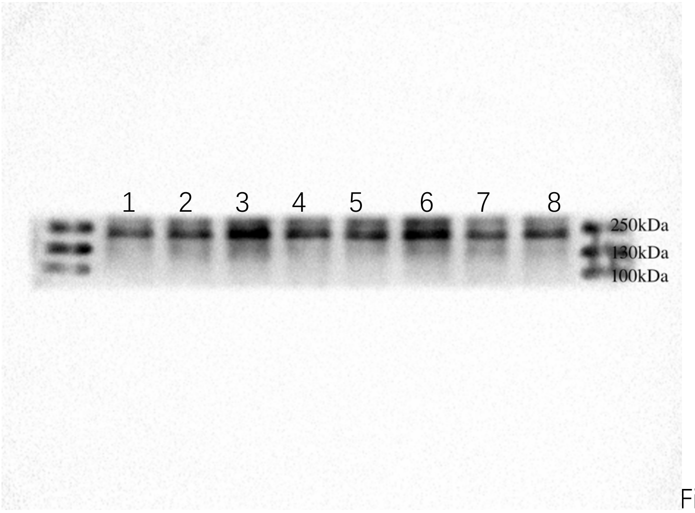

Fig7A GluN2A

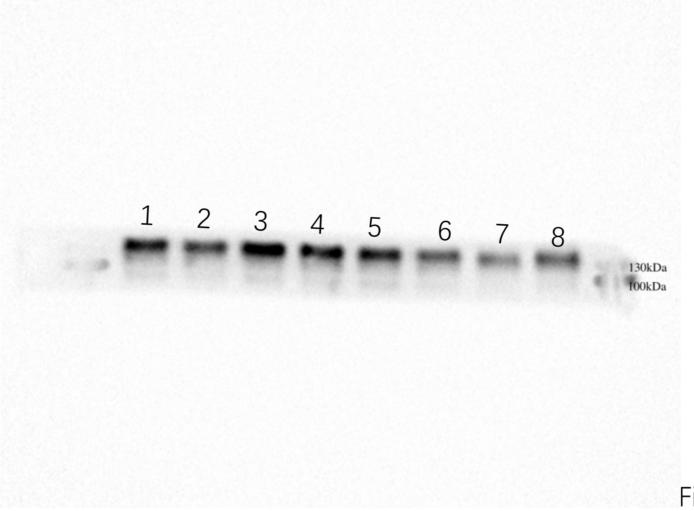

Fig7A p-GluN2A

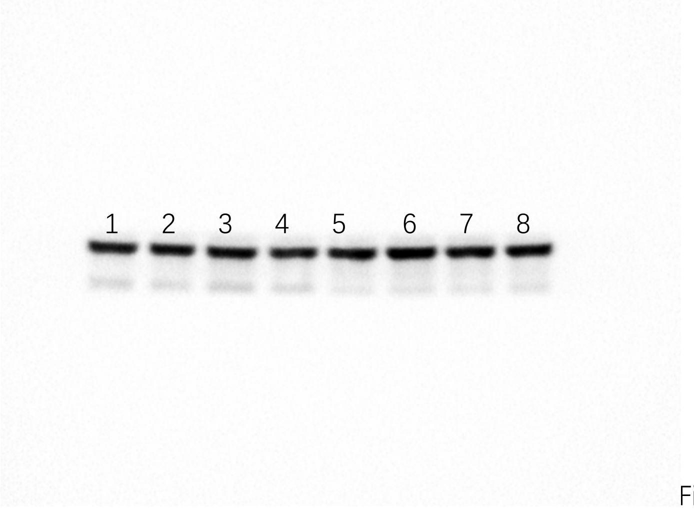

Fig7C β-actin

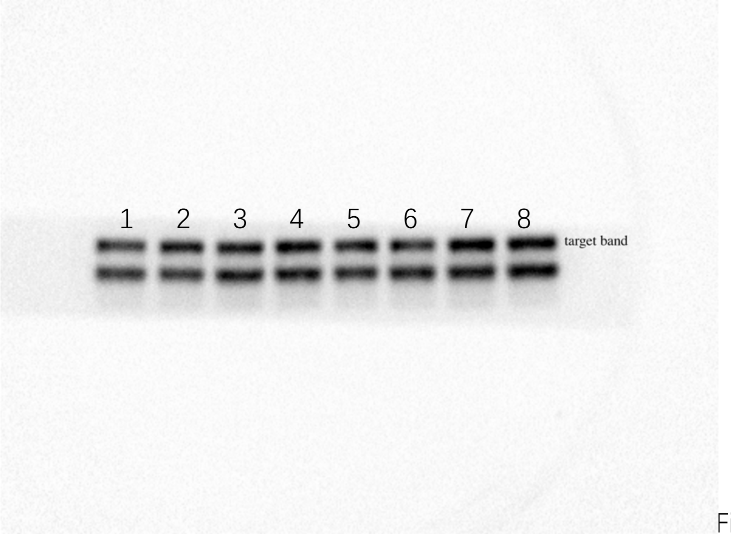

Fig7C GluN2B

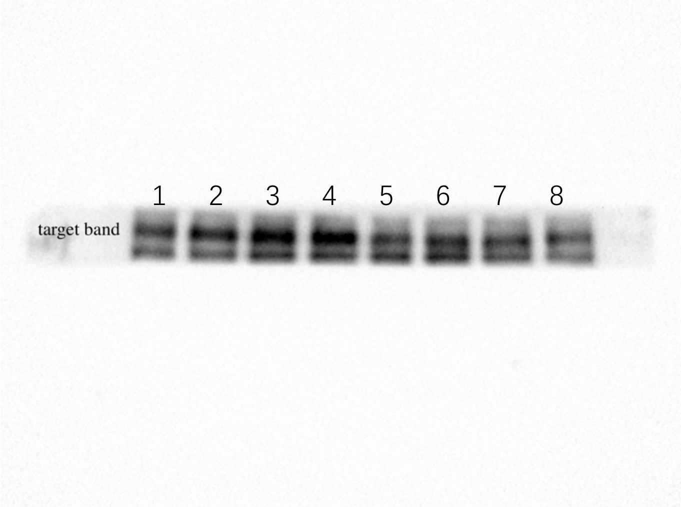

Fig7C p-GluN2B

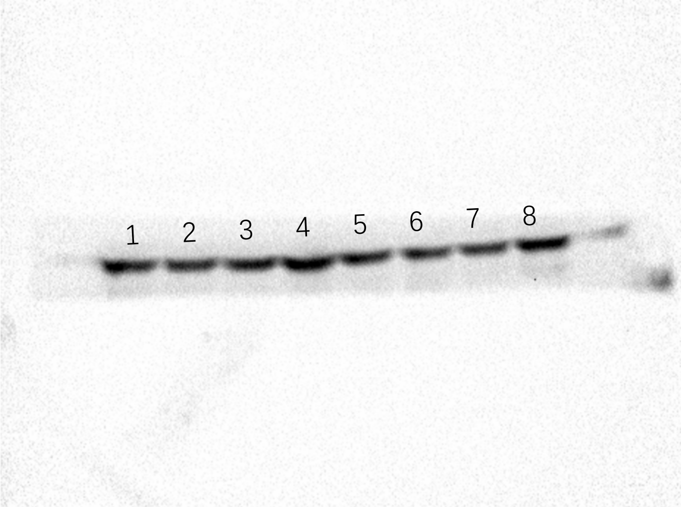

Fig7F fyn

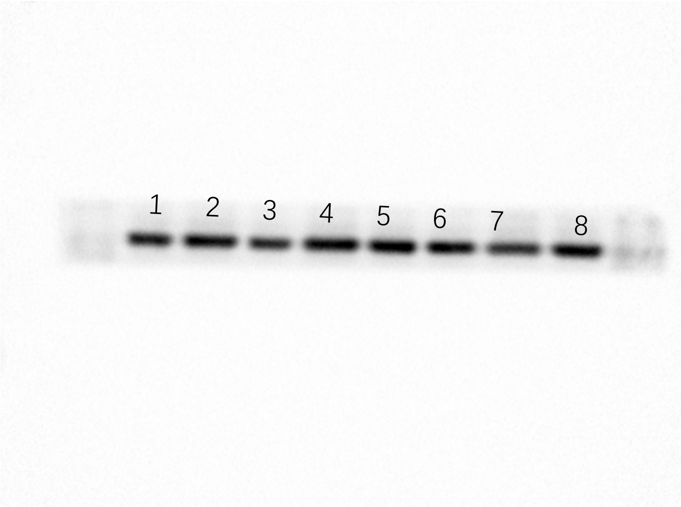

Fig7F GAPDH

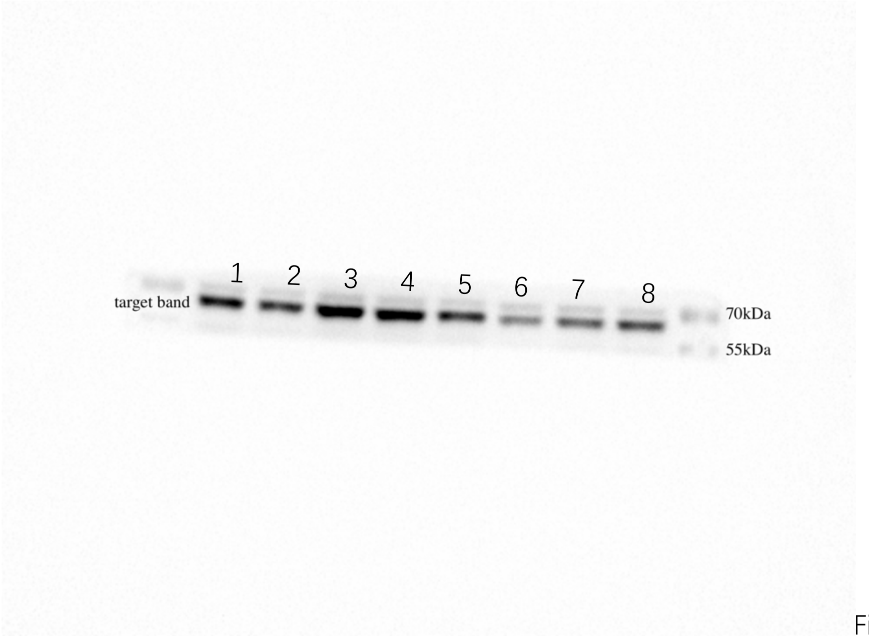

Fig7F p-fyn

1, 2-Normal + Saline group

3, 4- MPTP + Saline group

5, 6- MPTP + LY354740 0.5 mg/kg group

7, 8- MPTP + LY354740 4 mg/kg group

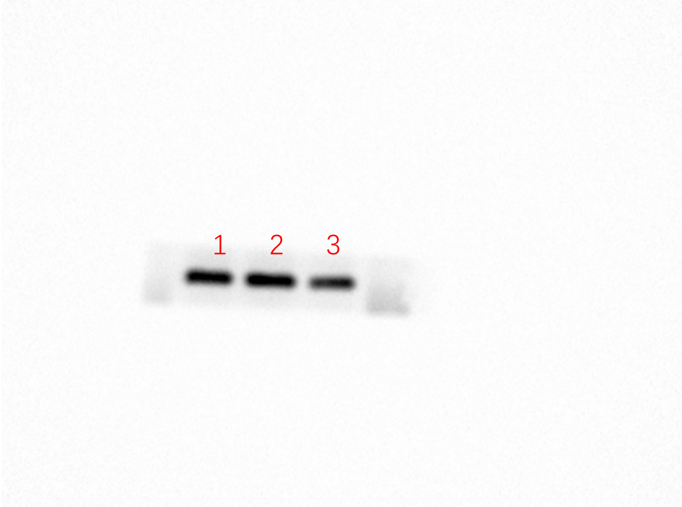

Fig7H GADPH

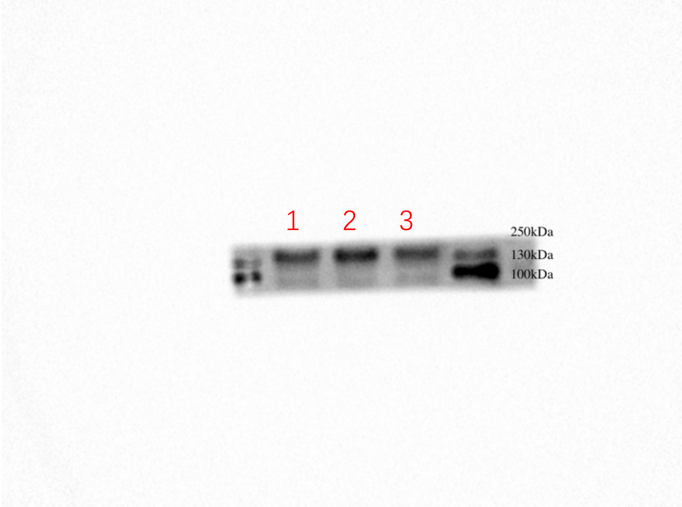

Fig7H GluN2A

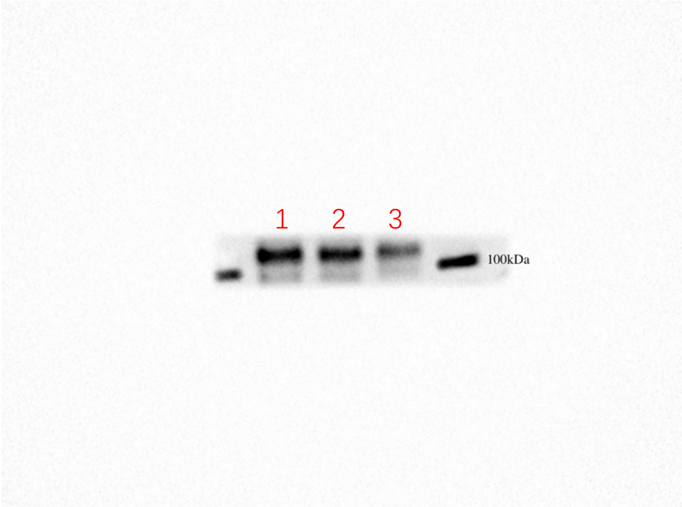

Fig7H p-GluN2A

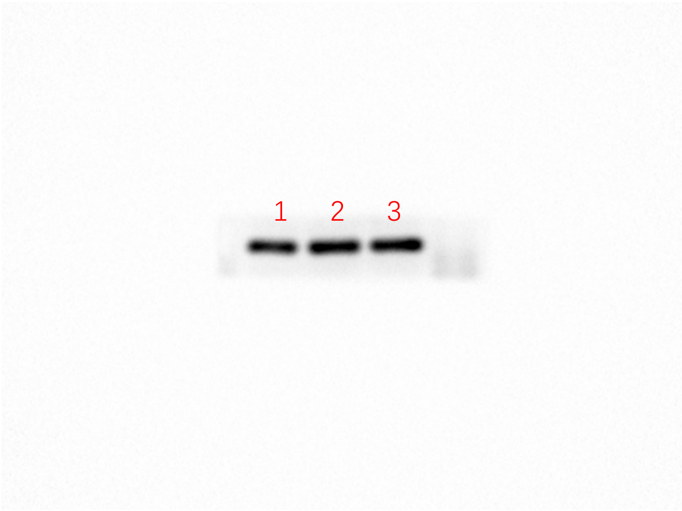

Fig7I GAPDH

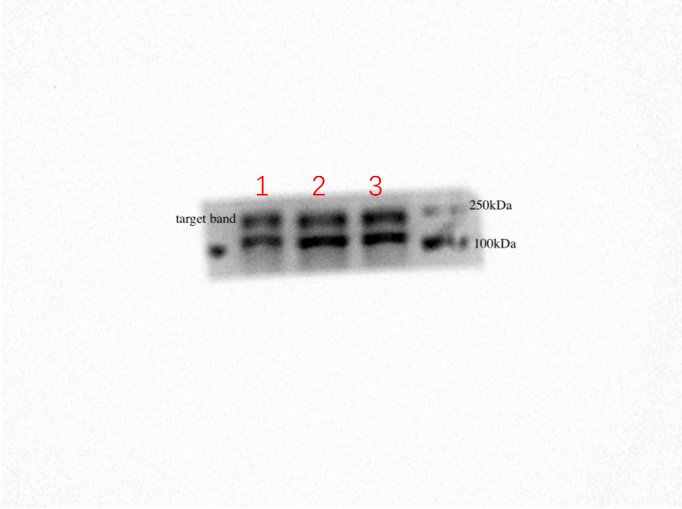

Fig7I GluN2B

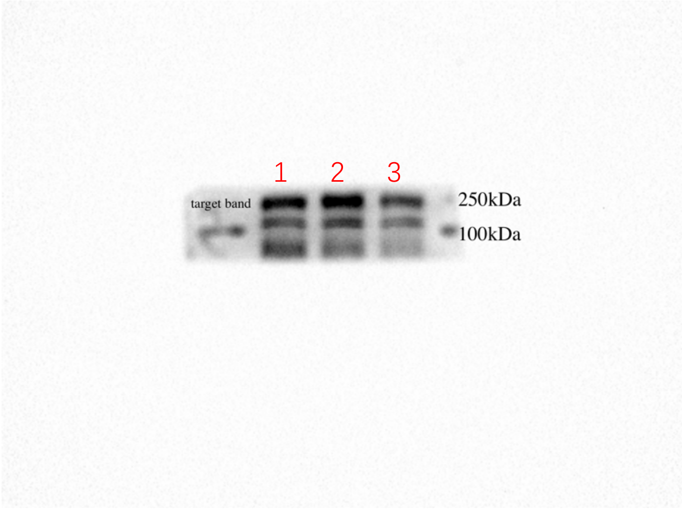

Fig7I p-GluN2B

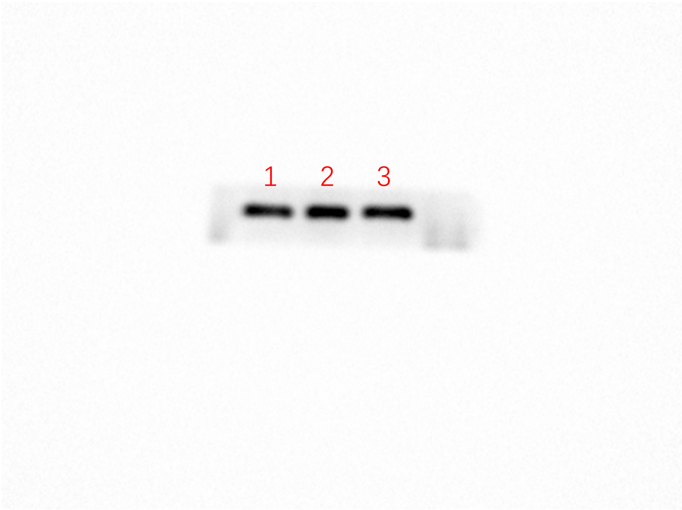

Fig7J Fyn

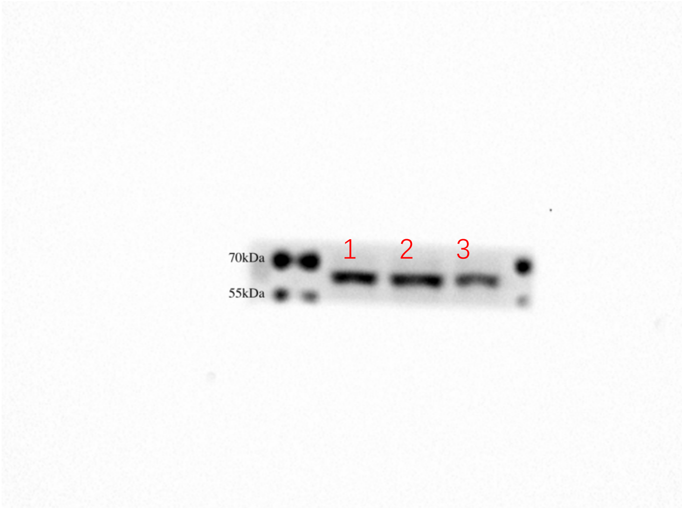

Fig7J p-Fyn

1-MPTP + Saline group

2-MPTP + LY341495 1 mg/kg group

3-MPTP + LY354740 4 mg/kg group

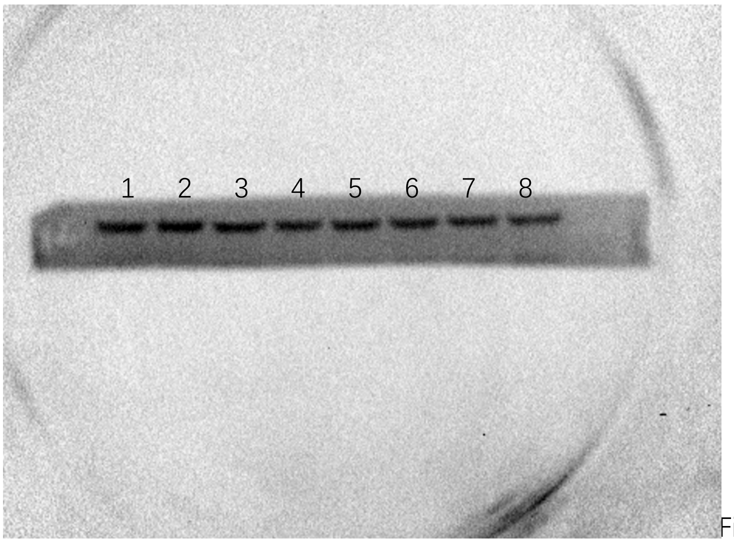

Fig8A β-actin

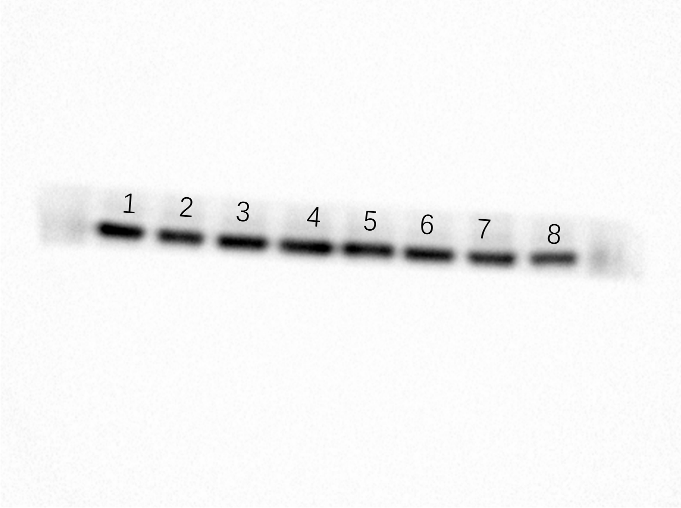

Fig8A GAPDH

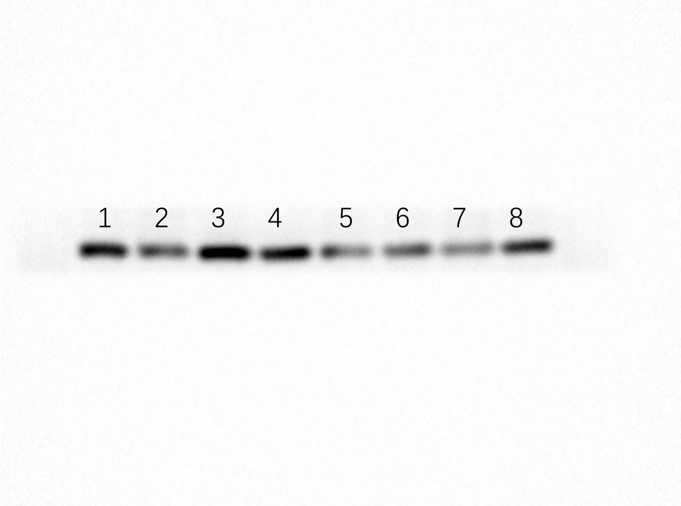

Fig8A PLK2

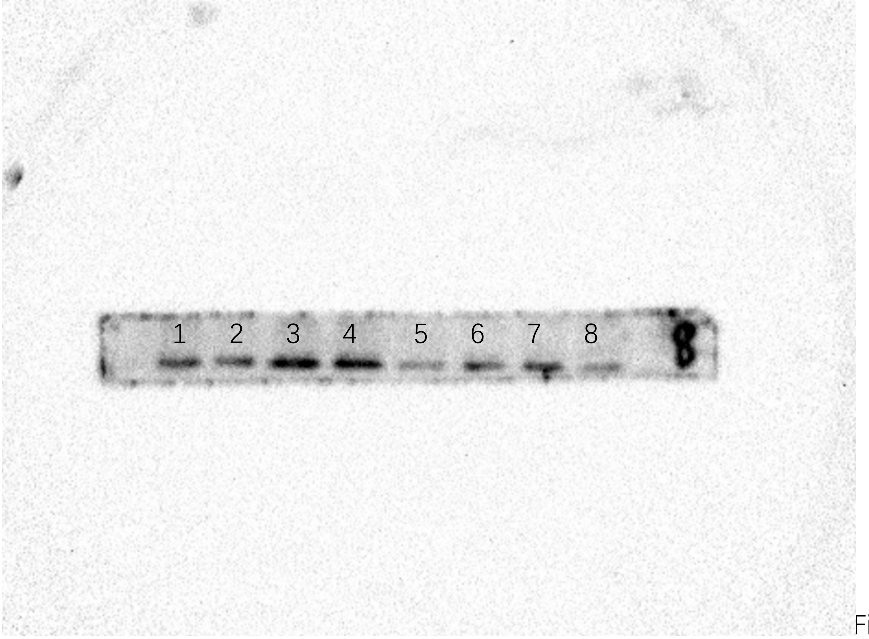

Fig8A p-syn

1, 2-Normal + Saline group

3, 4- MPTP + Saline group

5, 6- MPTP + LY354740 0.5 mg/kg group

7, 8- MPTP + LY354740 4 mg/kg group

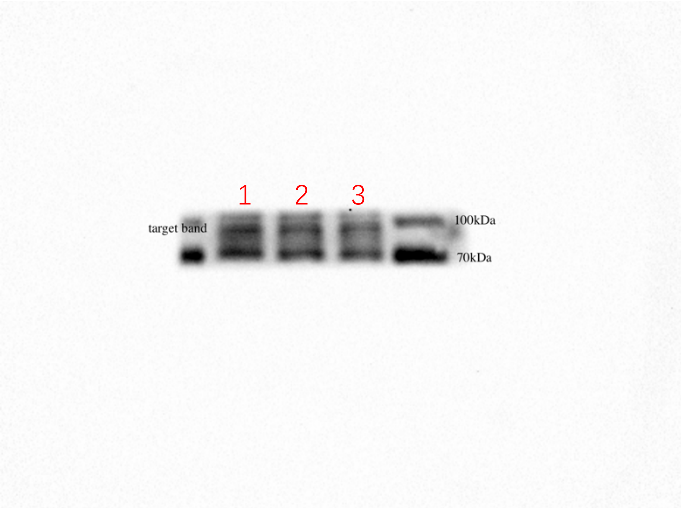

Fig8B PLK2

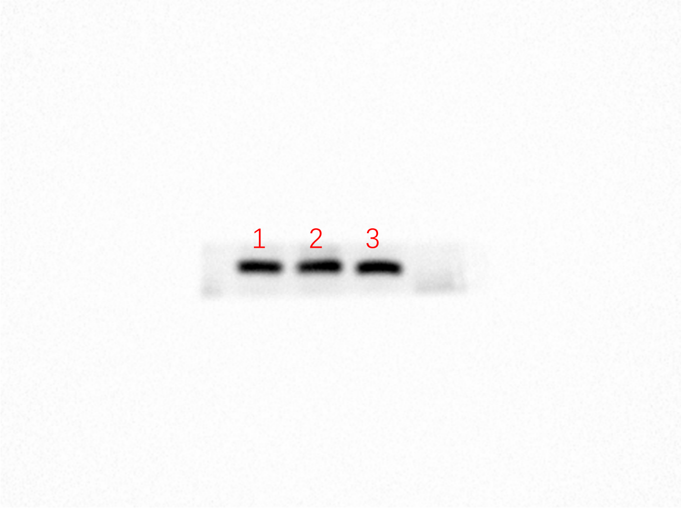

Fig8B GAPDH (PLK2)

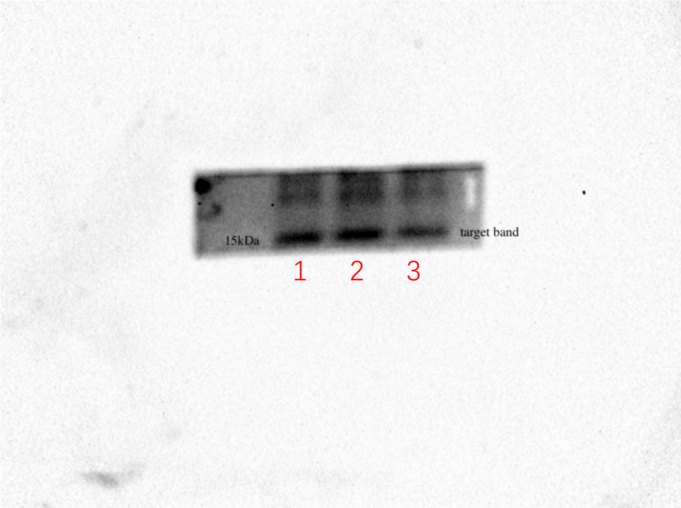

Fig8B p-syn

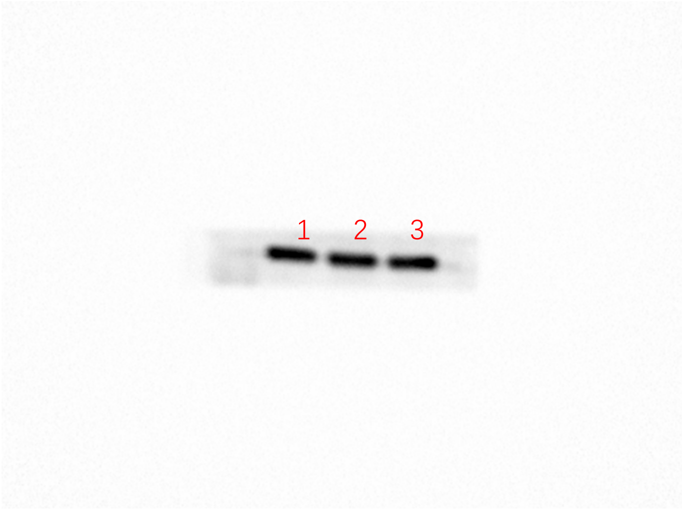

Fig8B GAPDH (p-syn)

1-MPTP + Saline group

2-MPTP + LY341495 1 mg/kg group

3-MPTP + LY354740 4 mg/kg group

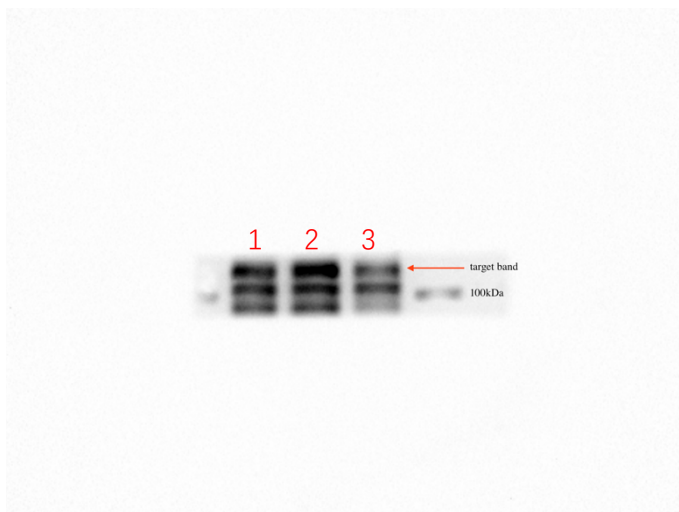

Supplement: Supplementary file 1 [file DataSheet_1.pdf]
